# A Multi-Criteria Framework for Comparative Evaluation and Robust Ranking of Fiber-Optic Gyroscopes

**DOI:** 10.3390/s26216838

**Published:** 2026-10-29

**Authors:** Nurzhigit Smailov, Yerlan Tashtay, Maral Abulkhanova, Daulet Naubetov, Kanat Zhunussov, Sauletbek Koshkinbayev, Beibarys Sekenov, Aziskhan Amir

**Affiliations:** 1 Department of Electronics, Telecommunications and Space Technologies, Satbayev University, Almaty 050013, Kazakhstan; n.smailov@satbayev.university (N.S.); m.abulkhanova@satbayev.university (M.A.); k.zhunussov@satbayev.university (K.Z.); b.sekenov@satbayev.university (B.S.); amirazishan@gmail.com (A.A.); 2 Department of Cybersecurity, Almaty University of Power Engineering and Telecommunications Named After Gumarbek Daukeyev, Almaty 050013, Kazakhstan; 3 Department of IT and Telecommunications, Miras University, Shymkent 160012, Kazakhstan; koshkinbaev_s@miras.edu.kz

**Keywords:** fiber-optic gyroscope, inertial navigation, multi-criteria decision-making, performance index, ranking robustness, normalization sensitivity, uncertainty analysis, FOGPI

## Abstract

Comparative evaluation of commercially available fiber-optic gyroscopes (FOGs) is complicated by heterogeneous technical specifications and the methodological assumptions underlying multi-criteria ranking. This study develops and systematically evaluates the Fiber Optic Gyroscope Performance Index (FOGPI), a six-criterion, specification-based framework integrating Bias Stability, Angle Random Walk, Maximum Input Rate, Bandwidth, Operating Temperature Range Width, and Power Consumption. The framework employs normalized technical characteristics and explicitly defined engineering preference weights and is applied to eleven commercial FOG models. Its methodological sensitivity is examined through linear, logarithmic, and fixed-reference normalization; local and global weight sensitivity analyses; mission-oriented Analytic Hierarchy Process (AHP) scenarios; intercriterion correlation and CRITIC weighting; leave-one-out analysis; hypothetical Monte Carlo input perturbations; and comparison with TOPSIS and VIKOR. Under baseline linear Min–Max normalization, DSP-1760 achieved the highest FOGPI score (0.816965). However, logarithmic normalization of accuracy-related criteria changed the highest-ranked device to S-F120HA, while increasing the Accuracy group weight beyond approximately 0.592 produced a similar rank reversal. Global weight-space analysis showed that DSP-1760 occupied first place in 50.72% of uniformly sampled configurations, compared with 87.39% under baseline-centered sampling. FOGPI and VIKOR produced identical rankings, although their agreement does not constitute independent validation because of their mathematical relationship. Application-specific screening further demonstrated that aggregate rankings cannot replace mandatory technical requirements. The findings establish FOGPI as a transparent decision-support framework whose rankings depend on normalization, weighting assumptions, comparison-set composition, and the comparability of manufacturer-reported specifications, rather than as an absolute measure of gyroscope performance.

## 1. Introduction

Modern inertial navigation systems (INSs) play an essential role in autonomous navigation for aviation, maritime transportation, robotics, unmanned aerial vehicles, and other applications in which satellite-based navigation may be unavailable, degraded, or unreliable. An INS estimates the position, velocity, and orientation of a moving platform by integrating measurements from gyroscopes and accelerometers. Gyroscopes provide angular-rate measurements required for attitude estimation, while their measurement errors contribute to the accumulation of navigation errors during autonomous operation [1,2].

Among the available angular-rate sensing technologies, fiber-optic gyroscopes (FOGs) are widely used in demanding inertial-navigation applications because they combine high measurement accuracy, the absence of mechanically moving sensing elements, and reliable operation over extended periods. These characteristics make FOGs suitable for navigation and stabilization systems requiring precise attitude estimation and operation under challenging environmental conditions [3,4].

Fiber-optic gyroscopes occupy an important position among optical and electromechanical angular-rate sensors, alongside microelectromechanical systems (MEMS) gyroscopes and ring laser gyroscopes (RLGs). Conventional interferometric fiber-optic gyroscopes (IFOGs) measure angular rotation through the Sagnac phase difference between counterpropagating optical waves in a fiber sensing coil [5]. In parallel with improvements in conventional IFOGs, research has increasingly explored miniaturized optical gyroscopes based on resonant and integrated photonic architectures. Planar photonic gyroscope concepts employing optical ring resonators, including Bragg-grating resonator configurations, have been investigated for satellite attitude-control applications, with particular attention to reducing size, mass, and power requirements [6,7]. These developments illustrate the potential of photonic integration for compact optical inertial sensing while also highlighting differences in measurement principles and implementation technologies.

Recent research has distinguished two principal categories of integrated optical gyroscopes: integrated interferometric optical gyroscopes (IIOGs) and integrated resonant optical gyroscopes (IROGs) [8]. IIOGs employ interferometric phase measurement, whereas IROGs exploit resonance-related optical responses to detect rotation. Integrated photonic implementations offer opportunities for miniaturization and component integration but introduce challenges involving optical losses, thermal sensitivity, noise, and fabrication-dependent performance. In resonant architectures, the resonator quality factor is an important parameter influencing sensing characteristics. For aerospace applications, environmental qualification and radiation tolerance are additional considerations, although these capabilities cannot be assumed solely from the use of integrated photonic technology. Accordingly, conventional IFOGs, integrated interferometric devices, and resonant optical gyroscopes should be assessed within the context of their respective architectures and application requirements.

The present study focuses on commercial FOG models with publicly documented technical specifications rather than directly comparing conventional interferometric, integrated photonic, and resonant gyroscope architectures. Within this scope, the literature identifies numerous characteristics relevant to gyroscope evaluation, including bias-related stability, angle random walk, scale-factor characteristics, dynamic response, and environmental operating conditions [9,10,11,12]. Bias Stability (BS) and Angle Random Walk (ARW) are frequently reported accuracy-related characteristics associated with low-frequency bias behavior and stochastic noise, respectively [13,14,15,16,17]. However, their definitions and measurement conditions may differ among manufacturers. Maximum Input Rate (MIR), Bandwidth (BW), Operating Temperature Range Width (TRW), and Power Consumption (PC) provide additional information about dynamic capability, specified operating conditions, and energy requirements. These characteristics describe different aspects of device performance and cannot be interpreted as interchangeable measures of gyroscope quality.

Existing studies have extensively investigated improvements in FOG architectures, error compensation, signal processing, and individual measurement characteristics, including bias-related errors, random noise, scale-factor behavior, and temperature-dependent performance [18,19,20,21]. Experimental characterization commonly involves Allan variance analysis, linearity assessment, dynamic testing, and evaluation under different environmental conditions [10,13,19]. Such investigations provide essential information about sensor behavior and measurement quality. However, the integration of heterogeneous manufacturer-reported characteristics into comparative engineering rankings introduces a different methodological problem: numerical scores may depend not only on the reported technical specifications but also on the normalization functions, weighting assumptions, and aggregation procedures adopted for the comparison.

Although numerous studies have addressed the development and characterization of fiber-optic gyroscopes [22,23,24,25,26,27,28,29,30,31,32], the methodological sensitivity of specification-based multi-criteria rankings warrants further systematic investigation. General-purpose multi-criteria decision-making (MCDM) approaches, including weighted aggregation, TOPSIS, and VIKOR, provide established tools for evaluating alternatives under multiple criteria [33,34,35]. Nevertheless, the resulting rankings may vary with criterion scaling, preference weights, intercriterion dependencies, and the composition of the comparison dataset. These issues are particularly relevant when the technical characteristics span substantially different numerical ranges or originate from manufacturer specifications obtained under heterogeneous reporting conditions. Therefore, the development and critical evaluation of a transparent, FOG-oriented comparative framework remain of practical and methodological interest [36].

The objective of this study is to develop and systematically investigate a specification-based multi-criteria framework for comparing selected characteristics of commercial fiber-optic gyroscopes. The proposed Fiber Optic Gyroscope Performance Index (FOGPI) combines six technical attributes—BS, ARW, MIR, BW, TRW, and PC—into a dimensionless comparative score using normalized criterion values and explicitly defined weighting coefficients. The index is intended to support structured engineering comparison within a specified decision context rather than provide an absolute measure of overall gyroscope performance or replace application-specific qualification.

The principal contribution of the study is the systematic examination of the methodological assumptions governing the interpretation and stability of FOGPI rankings. The framework does not introduce a fundamentally new mathematical aggregation operator; instead, it applies established normalization and weighted aggregation principles to a FOG-specific evaluation problem. Particular attention is given to the influence of linear and logarithmic normalization, sample-dependent and fixed-reference scaling, local and global weighting variations, intercriterion correlations, and hypothetical uncertainty in reported specifications. The analysis also distinguishes unrestricted compensatory ranking from application-specific admissibility screening, in which mandatory technical requirements must be satisfied before alternatives are ranked. Together, these investigations provide a basis for identifying conditions under which aggregate rankings are stable, sensitive, or potentially misleading.

The proposed framework is evaluated using eleven commercial FOG models representing heterogeneous accuracy, dynamic, operating-temperature, and power-consumption characteristics. Baseline FOGPI rankings are compared with TOPSIS and VIKOR using identical criteria and preference weights to examine methodological dependence rather than establish independent validation. Alternative normalization procedures are investigated to assess the influence of criterion scaling, while local weight sensitivity, mission-oriented AHP scenarios, and global weight-space sampling are used to examine preference dependence. Spearman correlation analysis and CRITIC weighting are employed to investigate intercriterion relationships and their influence on ranking outcomes. Leave-one-out analysis and Monte Carlo simulation are used to evaluate sensitivity to comparison-set composition and hypothetical input-specification variations, respectively. Finally, temperature-based and application-specific admissibility analyses illustrate how mandatory technical constraints can complement the comparative index. These analyses collectively characterize the methodological behavior, practical applicability, and limitations of the proposed FOGPI framework.

## 2. Materials and Methods

The proposed methodology comprises the selection and specification-based assessment of fiber-optic gyroscopes, definition of evaluation criteria, normalization and weighting of technical characteristics, and calculation of the Fiber Optic Gyroscope Performance Index (FOGPI). The framework is further examined through alternative normalization and weighting strategies, criterion-correlation analysis, uncertainty propagation, dataset-composition sensitivity, and comparison with established multi-criteria decision-making methods. Application-specific admissibility screening is additionally employed to distinguish mandatory technical requirements from preference-based ranking.

### 2.1. Selection of Fiber-Optic Gyroscopes

A comparative dataset of commercial FOG models with publicly documented technical specifications was compiled for the evaluation of the proposed methodology. The objective of the sampling procedure was not to provide an exhaustive survey of the global FOG market, but to assemble a heterogeneous set of devices spanning substantially different accuracy, dynamic, operating-temperature, and power-consumption characteristics.

The device search was based on publicly available technical documentation and product specifications collected during the preparation of the study. The dataset represents a documented selection of commercial FOG models rather than a verification of their market availability at the time of manuscript submission.

A FOG model was included in the dataset when the following criteria were satisfied:

- The device was identified as a FOG intended for navigation, stabilization, attitude determination, or related inertial applications;
- Quantitative Bias Stability (BS) and Angle Random Walk (ARW) specifications were available;
- Maximum Input Rate (MIR), Bandwidth (BW), Power Consumption (PC), and the minimum and maximum operating temperatures required to calculate Operating Temperature Range Width (TRW) could be established from the collected technical information;
- The available documentation provided sufficient information to construct the complete six-criterion decision matrix used in the comparative analysis.

Devices for which one or more of the six required numerical characteristics could not be established from the available sources were excluded from the decision matrix. No missing numerical values were estimated or imputed. However, the availability of a reported numerical specification was distinguished from its independent verification against model-specific technical documentation. For GF120H-A, the available model-specific documentation reports ARW ≤ 0.002°/√h, a maximum input rate of ±500°/s, and an operating-temperature interval of −40 to +65 °C. It also lists two zero-bias stability limits, ≤0.010°/h (1σ, 10 s) and ≤0.004°/h (1σ, 100 s). The latter value was retained in the decision matrix as a manufacturer-reported specification limit rather than an independently verified measurement. Bandwidth and power-consumption values could not be confirmed for the exact model configuration and were therefore retained as provisional inputs. These limitations were documented in the specification audit.

To assess the traceability and metrological comparability of the collected specifications, a document-based audit was performed. For each FOG, the available technical documentation was examined to identify the statistical definitions, measurement conditions, operating configurations, and specification types associated with BS, ARW, MIR, BW, and PC. Particular attention was given to measurement temperature, averaging time, signal-processing conditions, and whether the reported values represented typical performance, maximum limits, or other manufacturer-defined specifications.

The audit was guided by the specification and testing principles of IEEE Std 952-2020 [37] for single-axis interferometric FOGs. The standard was used as a methodological reference for assessing the completeness and comparability of the published specifications. Compliance with IEEE Std 952-2020 was not assumed unless explicitly documented by the manufacturer.

Where the available documentation did not specify a measurement condition or statistical definition, the corresponding information was recorded as not reported (NR). No undocumented measurement conditions were inferred. The results of this assessment are presented in Section 3.1.2.

For descriptive purposes, the investigated devices were grouped into navigation-oriented, tactical-oriented, and compact categories based on their reported characteristics, intended applications, and product configurations. These labels are qualitative descriptors of the selected sample rather than standardized performance classifications defined by numerical thresholds. The S-F120HA, S-F120HB, S-F120HC, GF120H-A, and EG-1300 models were assigned to the navigation-oriented category because of their comparatively low Bias Stability and Angle Random Walk specifications and their intended high-accuracy navigation applications [38,39,40].

DSP-1760, DSP-3000, and EG-200 were assigned to the tactical-oriented category based on their reported performance characteristics and intended navigation, stabilization, and control applications [41,42,43]. VG103SD, VG1703, and VG910F1 were categorized as compact FOGs because their product specifications emphasize compact implementation, low power consumption, and suitability for size- and energy-constrained platforms [44,45,46].

These class labels are descriptive and are not used as evaluation criteria, weighting factors, or constraints in the calculation of FOGPI. Accordingly, the numerical ranking is determined exclusively from the six quantitative characteristics included in the decision matrix.

Power Consumption (PC) was evaluated using the values reported for individual FOG units or explicitly specified single-axis configurations rather than complete multi-axis inertial measurement units. Because the source documentation includes typical, maximum, steady-state, and unspecified power-consumption values, the collected PC characteristics are not necessarily metrologically equivalent. These differences were documented in the specification audit and considered when interpreting the comparative results.

The resulting dataset comprises eleven FOGs with substantially different performance profiles. Although the sample provides sufficient heterogeneity for investigating the behavior of the proposed comparative framework, it should not be interpreted as a statistically representative or exhaustive sample of commercial FOG models. The technical characteristics, manufacturer information, and corresponding data sources for the investigated FOGs are provided in Table 1 to ensure traceability of the specifications used in the comparative analysis.

### 2.2. Criteria for Evaluating the Characteristics of Fiber-Optic Gyroscopes

To develop the proposed multi-criteria evaluation framework, six technical characteristics were selected to represent specific accuracy-related, dynamic, operating-temperature, and energy-related aspects of FOG operation relevant to inertial navigation applications. The criteria were selected taking into account their availability in technical documentation, the possibility of quantitative comparison across different models, and their significance for evaluating gyroscopic sensor performance [48,49,50,51].

The term Bias Stability (BS) is used throughout this study as a common label for the bias-related characteristic reported in the corresponding manufacturer documentation. However, the original specifications may refer to bias stability, bias instability, in-run bias stability, or other related quantities determined using different statistical definitions and measurement procedures. These terms are not assumed to be metrologically equivalent, and the original reporting conditions are documented in Section 3.1.

As a result, six criteria were used for further analysis: Bias Stability (BS), Angle Random Walk (ARW), Maximum Input Rate (MIR), Bandwidth (BW), Operating Temperature Range Width (TRW) and Power Consumption (PC). Together, these parameters represent accuracy-related, dynamic, operating-temperature, and energy-related characteristics of the investigated FOGs.

For subsequent calculation of the Fiber Optic Gyroscope Performance Index (FOGPI), the selected criteria were organized into four functional groups:

- Accuracy—Bias Stability and Angle Random Walk;
- Dynamic Performance—Maximum Input Rate and Bandwidth;
- Operating Temperature Capability—Operating Temperature Range Width;
- Power Requirement—Power Consumption.

The rationale for selecting each criterion and its role in the proposed evaluation methodology are presented in Section 2.2.1, Section 2.2.2, Section 2.2.3 and Section 2.2.4.

#### 2.2.1. Accuracy

The Accuracy group in the proposed framework represents two selected error-related characteristics rather than a comprehensive measure of gyroscope accuracy. Bias Stability (BS) and Angle Random Walk (ARW) were selected because bias-related errors and stochastic noise contribute to navigation-error accumulation during autonomous inertial operation [52].

Bias Stability (BS) characterizes the stability of the gyroscope bias under specified operating and measurement conditions and reflects low-frequency variations in the zero-rate output. This parameter is important because uncompensated bias contributes to the accumulation of navigation errors during autonomous operation of an inertial navigation system [53]. The BS value is expressed in degrees per hour (°/h).

Angle Random Walk (ARW) characterizes the level of random noise of the gyroscope output signal and represents the random noise component that accumulates during angular-rate integration [54]. This indicator is widely used to evaluate the short-term accuracy of gyroscopic sensors and is measured in degrees per square root hour (°/√h).

Since lower values of BS and ARW correspond to more favorable accuracy characteristics, both parameters were treated as cost criteria (“lower is better”) in the normalization procedure described in Section 2.3.

#### 2.2.2. Dynamic Performance

Dynamic characteristics describe the specified operating capabilities of a FOG under varying angular-rate conditions. In this study, two parameters were selected to represent these capabilities: Maximum Input Rate (MIR) and Bandwidth (BW) [55].

Maximum Input Rate (MIR) represents the maximum angular rate specified for measurement by the gyroscope. This parameter is particularly relevant to highly maneuverable platforms, including unmanned aerial vehicles, robotic systems, and vehicles in which high angular rates may occur. MIR is expressed in degrees per second (°/s).

Bandwidth (BW) characterizes the frequency range over which a gyroscope can respond to variations in angular rate and is therefore relevant to the measurement of rapidly changing rotational motion [56]. A higher bandwidth generally allows the sensor to respond to faster variations in angular rate, although the resulting measurement accuracy also depends on noise, filtering, and other sensor characteristics [57]. BW is expressed in hertz (Hz).

Within the selected comparative framework, higher MIR and BW values were considered preferable because they represent broader specified angular-rate and frequency-response capabilities. Accordingly, both parameters were treated as benefit criteria in the normalization procedure described in Section 2.3.

#### 2.2.3. Operating Temperature Capability

The operating temperature interval specifies the minimum and maximum temperatures within which a FOG is rated by the manufacturer for operation. Because operating-temperature specifications were consistently available for the investigated devices, they were included as an operational criterion in the comparative evaluation [8,58,59].

For quantitative comparison, the Operating Temperature Range Width (TRW) was calculated as:

*TRW* = *T_max_* − *T_min_* (1)

where *T_max_* and *T_min_* are maximum and minimum specified operating temperatures.

A larger TRW indicates that the device is specified for operation over a broader temperature interval and was therefore treated as a benefit criterion. However, TRW represents only the width of the operating-temperature interval and does not indicate its absolute lower and upper limits. Furthermore, the manufacturer’s specified operating range does not establish that Bias Stability, Angle Random Walk, scale-factor characteristics, or other performance parameters remain constant throughout that interval. A comprehensive assessment of environmental suitability would require additional information on temperature-dependent accuracy, vibration and shock resistance, humidity, magnetic sensitivity, and radiation tolerance. These characteristics were not included in the present index because sufficiently comparable specifications were unavailable for all investigated devices.

Therefore, the absolute minimum and maximum operating-temperature limits were evaluated separately as mandatory constraints in the application-specific admissibility analysis described in Section 2.13, rather than being inferred from TRW alone.

#### 2.2.4. Power Consumption

Power Consumption (PC) was included as an energy-related criterion because it is commonly reported in manufacturers’ specifications and directly affects the integration of FOGs into energy-constrained systems. PC represents the electrical power required for gyroscope operation and is expressed in watts (W).

Lower power consumption reduces the demand placed on the onboard power supply and may be particularly advantageous for battery-powered or energy-constrained platforms, including unmanned aerial vehicles and mobile robotic systems. Accordingly, PC was treated as a cost criterion, for which lower values correspond to a more favorable evaluation.

Because the reported PC values were obtained under different or incompletely specified operating conditions, this criterion was treated as a specification-based comparative attribute rather than a standardized measure of power consumption, as discussed in Section 2.1.

The six evaluation criteria were selected to represent a limited set of accuracy-related, dynamic, operating-temperature, and power-related characteristics available for the investigated FOGs. They do not constitute an exhaustive description of gyroscope performance. Additional characteristics, including scale-factor stability and nonlinearity, bias repeatability, acceleration sensitivity, vibration and shock resistance, magnetic sensitivity, radiation tolerance, long-term reliability, mean time between failures (MTBF), startup and warm-up time, mass, volume, dimensions, and communication interfaces, may be critical for specific navigation, stabilization, or aerospace applications. These parameters were excluded from the common quantitative decision matrix because sufficiently complete and consistently defined specifications were not available across all investigated devices. Consequently, FOGPI should be interpreted as a six-criterion, specification-based comparative index rather than a comprehensive measure of overall gyroscope performance.

### 2.3. Data Preprocessing and Normalization

The technical characteristics of the investigated FOGs differ in their physical quantities, units of measurement, and numerical ranges. Therefore, they cannot be directly combined to calculate the proposed Fiber Optic Gyroscope Performance Index (FOGPI).

To ensure comparability among the criteria, the initial data were normalized using Min–Max normalization, which transforms all criteria into a common dimensionless range from 0 to 1 while preserving their ordering and linear relative spacing within each criterion for a fixed comparison dataset. Min–Max normalization was selected because of its transparency and straightforward interpretation, making it suitable for the subsequent weighted aggregation. However, the resulting normalized values depend on the minimum and maximum values of the comparison dataset; therefore, they should be interpreted as relative rather than intrinsic characteristics of the evaluated FOGs.

The following expression was used for criteria whose values should be minimal to ensure the best performance (Bias Stability, Angle Random Walk, and Power Consumption):

(2) $$X_{i}^{\mathrm{norm}}=\frac{X_{\max}-X_{i}}{X_{\max}-X_{\min}}$$

where *X_i_* is the original value of the considered criterion for the *i*-th gyroscope; *X_max_* and *X_min_* are the maximum and minimum values of this criterion within the comparison dataset, respectively; and $X_{i}^{\mathrm{norm}}$ is the normalized value of the criterion.

For criteria whose values should be maximum (Maximum Input Rate, Bandwidth, and Operating Temperature Range Width), the following dependence was applied:

(3) $$X_{i}^{\mathrm{norm}}=\frac{X_{i}-X_{\min}}{X_{\max}-X_{\min}}$$

where all variables are defined as above.

If all alternatives have identical values for a particular criterion, the normalized value is set to 1 for all devices, since that criterion does not discriminate among the alternatives. No such cases occurred in the investigated dataset.

After normalization, all six criteria were expressed on a common dimensionless scale, where 1 represents the most favorable observed value and 0 the least favorable observed value for each criterion. These normalized values were subsequently used in the weighted aggregation procedure described in Section 2.5.

Although linear Min–Max normalization provides a transparent baseline, its use for BS and ARW may compress multiplicative performance differences when the reported values span several orders of magnitude. Furthermore, the normalization bounds depend on the composition of the comparison dataset. To examine these limitations, additional analyses were conducted using logarithmic normalization of BS and ARW and fixed-reference normalization with predefined criterion bounds. Both normalization approaches are described in Section 2.7.

### 2.4. Determination of Weighting Factors

After normalizing the evaluation criteria to a common dimensionless scale, weighting coefficients were assigned to represent their relative importance in the calculation of FOGPI. The present study adopts a baseline engineering preference scenario in which accuracy-related characteristics receive the greatest importance, reflecting their relevance to autonomous inertial navigation [60,61,62]. The numerical coefficients were selected as illustrative engineering assumptions rather than derived from expert elicitation, experimentally measured navigation-error budgets, or formally validated mission requirements. Accordingly, they are not intended to represent universal or objectively optimal weights.

The baseline scenario assigns the highest priority to the Accuracy group because bias-related errors and stochastic noise contribute to navigation-error accumulation during autonomous inertial operation. Accordingly, Accuracy was assigned a group weight of 0.50. Dynamic Performance was assigned a weight of 0.25 because the ability to measure rapid angular motion is important for maneuvering platforms, although its influence on long-term navigation error differs from that of accuracy-related error characteristics.

The Operating Temperature Capability group was assigned a weight of 0.15 to account for the specified operating-temperature range of the evaluated devices. The Power Requirement group was assigned a weight of 0.10 because power consumption primarily affects system integration and energy requirements rather than measurement accuracy itself.

The influence of the adopted weighting configuration on FOGPI scores and rankings is examined through local weight sensitivity analysis, mission-oriented AHP scenarios, global weight-space sampling, and alternative CRITIC-derived criterion weights, as described in the subsequent methodological sections. These complementary analyses characterize the dependence of the ranking on weighting assumptions rather than validate the baseline coefficients.

The baseline weighting coefficients assigned to the four functional groups are presented in Table 2.

The weighting coefficients were defined at the level of functional groups rather than directly for individual criteria. For groups containing more than one criterion, the corresponding group weight was distributed equally among the constituent criteria. This approach preserves the predefined contribution of each functional group to the overall weighting structure.

The resulting weighting coefficients for the individual criteria are presented in Table 3.

The resulting individual criterion weights sum to unity and preserve the relative importance assigned to the four functional groups. Equal allocation within the Accuracy and Dynamic Performance groups was adopted as a simplifying assumption rather than as evidence that the constituent criteria have identical physical significance. The baseline weighting configuration is subsequently used as the reference case for the comparative evaluation and sensitivity analyses.

### 2.5. Development of the Fiber Optic Gyroscope Performance Index (FOGPI)

This study proposes the Fiber Optic Gyroscope Performance Index (FOGPI) as a six-criterion, specification-based index for the comparative evaluation of selected technical characteristics of fiber-optic gyroscopes.

Because all criteria are normalized to the common interval [0, 1], FOGPI is calculated as a weighted linear combination of normalized characteristics. This approach provides a transparent and computationally reproducible procedure and allows the weighting structure to be adapted for different navigation applications.

The proposed methodology first calculates four group scores: Accuracy (*A*), Dynamic Performance (*D*), Operating Temperature Capability (*T*), and Power Requirement (*P*).

The Accuracy group score, representing the combined contribution of the two selected accuracy-related criteria, is calculated as:

(4) $$A=\frac{{\mathrm{BS}}_{n}+{\mathrm{ARW}}_{n}}{2}$$

where *BS_n_* and *ARW_n_* are the normalized values of the Bias Stability and the Angle Random Walk (ARW), respectively.

The dynamic performance indicator is defined as follows:

(5) $$D=\frac{{\mathrm{MIR}}_{n}+{\mathrm{BW}}_{n}}{2}$$

where *MIR_n_* and *BW_n_* are the normalized values of Maximum Input Rate and Bandwidth, respectively.

Since the Operating Temperature Capability and Power Requirement groups are represented by a single parameter, their values are defined directly as:

*T* = *TRW_n_* (6)

*P* = *PC_n_* (7)

where *TRW_n_* is the normalized Operating Temperature Range Width and *PC_n_* is the normalized Power Consumption.

*FOGPI* = *w_A_A* + *w_D_D* + *w_T_T* + *w_P_P* (8)

where *w_A_*, *w_D_*, *w_T_*, and *w_P_* are the weighting coefficients of the Accuracy, Dynamic Performance, Operating Temperature Capability, and Power Requirement groups, respectively, satisfying

*w_A_* + *w_D_* + *w_T_* + *w_P_* = 1 (9)

All weighting coefficients are non-negative, ensuring that the aggregate index remains within the normalized interval when the group scores lie between 0 and 1.

For the baseline weighting configuration adopted in this study, the general FOGPI expression becomes:

*FOGPI* = 0.50*A* + 0.25*D* + 0.15*T* + 0.10*P* (10)

The resulting FOGPI value lies within the interval [0, 1], with higher values indicating a more favorable aggregate score under the selected criteria, normalization procedure, and weighting configuration. FOGPI is a specification-based comparative index rather than an absolute measure of gyroscope quality. Its scores and rankings depend on the investigated alternatives and methodological assumptions and should therefore be interpreted within a defined comparison context rather than as universally valid indicators of intrinsic gyroscope performance.

Weighted linear aggregation was adopted because it provides a transparent, reproducible, and computationally efficient procedure for combining heterogeneous evaluation criteria [63,64]. However, its compensatory structure allows favorable performance in one criterion to offset weaker performance in another. Consequently, the resulting FOGPI ranking should be complemented by application-specific admissibility screening when mandatory technical requirements must be satisfied, as described in Section 2.13.

### 2.6. Benchmarking Against Established MCDM Methods

To investigate the sensitivity of the resulting rankings to the selected multi-criteria aggregation method, the eleven FOGs were additionally evaluated using the Technique for Order Preference by Similarity to Ideal Solution (TOPSIS) and VIKOR. These methods represent different decision-making principles: TOPSIS evaluates alternatives according to their relative distances from positive and negative ideal solutions, whereas VIKOR combines a group-utility measure with a maximum individual-regret measure to construct a compromise ranking. The comparison was intended to examine methodological dependence rather than provide independent validation of FOGPI.

For methodological consistency, all three approaches were applied to the same decision matrix comprising Bias Stability (BS), Angle Random Walk (ARW), Maximum Input Rate (MIR), Bandwidth (BW), Operating Temperature Range Width (TRW), and Power Consumption (PC). The weighting coefficients were fixed at 0.25, 0.25, 0.125, 0.125, 0.15, and 0.10 for BS, ARW, MIR, BW, TRW, and PC, respectively. BS, ARW, and PC were treated as cost criteria, for which lower values are preferable, whereas MIR, BW, and TRW were treated as benefit criteria.

For the TOPSIS analysis, the initial decision matrix was first transformed using vector normalization:

(11) $$r_{\mathrm{ij}}=\frac{x_{\mathrm{ij}}}{\sqrt{\sum_{i=1}^{m}x_{\mathrm{ij}}^{2}}}$$

where *x_ij_* is the value of criterion *j* for the *i*-th gyroscope, *r_ij_* is the corresponding vector-normalized value, and *m* is the number of alternatives. The weighted normalized decision matrix was then calculated as

*v_ij_* = *w_j_r_ij_* (12)

where *w_j_* denotes the weight assigned to criterion *j*.

The positive ideal solution *A*^+^ and negative ideal solution *A*^−^ were defined as

(13) $$A^{+}=\left\{v_{1}^{+},v_{2}^{+},\ldots ,v_{n}^{+}\right\}$$

(14) $$A^{-}=\left\{v_{1}^{-},v_{2}^{-},\ldots ,v_{n}^{-}\right\}$$

where, for benefit criteria

(15) $$v_{j}^{+}=\underset{i}{\max v_{\mathrm{ij}}},{\;v}_{j}^{-}=\underset{i}{\min v_{\mathrm{ij}}}$$

and, for cost criteria

(16) $$v_{j}^{+}=\underset{i}{\min v_{\mathrm{ij}}},{\;v}_{j}^{-}=\underset{i}{\max v_{\mathrm{ij}}}$$

The Euclidean distances of each alternative from the positive and negative ideal solutions were calculated as

(17) $$S_{i}^{+}=\sqrt{\sum_{j=1}^{n}{\left(v_{\mathrm{ij}}-v_{j}^{+}\right)}^{2}}$$

(18) $$S_{i}^{-}=\sqrt{\sum_{j=1}^{n}{\left(v_{\mathrm{ij}}-v_{j}^{-}\right)}^{2}}$$

The TOPSIS relative closeness coefficient was subsequently determined as

(19) $$C_{i}=\frac{S_{i}^{-}}{S_{i}^{+}+S_{i}^{-}}$$

A higher *C_i_* value indicates an alternative located closer to the positive ideal solution and therefore corresponds to a higher rank.

For the VIKOR analysis, the best $f_{j}^{*}$ and worst $f_{j}^{-}$ values were first identified for each criterion according to its benefit or cost orientation:

(20) $$f_{j}^{*}=\left\{\begin{array}{cc} \max_{i}f_{\mathrm{ij}}, & j\in J_{\mathrm{benefit}}\\ \min_{i}f_{\mathrm{ij}}, & j\in J_{\mathrm{cost}} \end{array}\right.$$

(21) $$f_{j}^{*}=\left\{\begin{array}{cc} \min_{i}f_{\mathrm{ij}}, & j\in J_{\mathrm{benefit}}\\ \max_{i}f_{\mathrm{ij}}, & j\in J_{\mathrm{cost}} \end{array}\right.$$

The group utility measure *S_i_* and individual regret measure *R_i_* were then calculated as

(22) $$S_{i}=\sum_{j=1}^{n}w_{j}\frac{f_{j}^{*}-f_{\mathrm{ij}}}{f_{j}^{*}-f_{j}^{-}}$$

(23) $$R_{i}=\underset{j}{\max}\left[w_{j}\frac{f_{j}^{*}-f_{\mathrm{ij}}}{f_{j}^{*}-f_{j}^{-}}\right]$$

For both benefit and cost criteria, the normalized deviation from the best criterion value was calculated using the corresponding best and worst values. Under this formulation, a deviation of zero represents the most favorable criterion value, whereas a deviation of one represents the least favorable value in the comparison dataset.

The compromise index *Q_i_* was calculated according to

(24) $$Q_{i}=v\frac{S_{i}-S^{*}}{S^{-}-S^{*}}+\left(1-v\right)\frac{R_{i}-R^{*}}{R^{-}-R^{*}}$$

where

(25) $$S^{*}=\underset{i}{\min S_{i},S^{-}}=\underset{i}{\max S_{i}}$$

(26) $$R^{*}=\underset{i}{\min R_{i},R^{-}}=\underset{i}{\max R_{i}}$$

The compromise parameter was set to *v* = 0.5, assigning equal importance to group utility and individual regret. Alternatives were ranked in ascending order of *Q_i_*, with lower values indicating solutions closer to the compromise optimum.

Under the adopted linear Min–Max normalization and identical criterion weights, the normalized criterion-level deviation used in the VIKOR group-utility measure is equal to one minus the corresponding benefit-oriented normalized value used in FOGPI. Since the criterion weights sum to unity, the relationship *S_i_* = 1 − *FOGPI_i_* holds for the investigated decision matrix. Consequently, the rankings obtained using FOGPI and the VIKOR group-utility measure *S_i_* are mathematically equivalent. However, the final VIKOR compromise index *Q_i_* also incorporates the maximum individual-regret measure *R_i_*. Therefore, identical complete rankings are not guaranteed for *v* = 0.5, and the observed agreement between FOGPI and VIKOR should be interpreted as conditional on the investigated dataset and weighting configuration.

Finally, the similarity between the rankings obtained using FOGPI, TOPSIS, and VIKOR was quantified using Spearman’s rank correlation coefficient and Kendall’s rank correlation coefficient. Spearman’s coefficient was calculated as

(27) $$\rho =1-\frac{6\sum_{i=1}^{m}d_{i}^{2}}{m\left(m^{2}-1\right)}$$

where *d_i_* is the difference between the ranks assigned to alternative *i* by the two compared methods and *m* is the number of ranked alternatives.

Kendall’s rank correlation coefficient was calculated as

(28) $$\tau =\frac{N_{c}-N_{d}}{m\left(m-1\right)/2}$$

where *N_c_* and *N_d_* are the numbers of concordant and discordant pairs, respectively.

The rank-correlation coefficients were calculated for complete rankings without tied positions; therefore, the standard no-tie formulations of Spearman’s and Kendall’s coefficients were applicable.

The resulting scores, rankings, and rank-correlation coefficients were used to characterize the dependence of the comparative evaluation on the selected decision-making method. Particular attention was given to differences in the positions of individual FOGs and to the interpretation of agreement between methods sharing the same decision matrix and weighting configuration. The corresponding results are presented in Section 3.7.

### 2.7. Alternative Normalization and Fixed-Reference Assessment

To investigate the influence of normalization assumptions on FOGPI scores and rankings, two complementary analyses were conducted. The first examined the effect of replacing linear Min–Max normalization with logarithmic normalization for the accuracy-related criteria, Bias Stability (BS) and Angle Random Walk (ARW). The second investigated fixed-reference normalization using predefined criterion bounds to reduce the dependence of numerical scores on the composition of the comparison dataset.

#### 2.7.1. Logarithmic Normalization of Accuracy-Related Criteria

The baseline FOGPI formulation employs linear Min–Max normalization, as described in Section 2.3. However, linear normalization may compress relative differences between high-accuracy devices when BS and ARW values span several orders of magnitude. To examine this limitation, logarithmic Min–Max normalization was applied separately to BS and ARW:

(29) $$n_{\mathrm{ij}}^{\log}=\frac{\ln\left(x_{j}^{\max}\right)-\ln\left(x_{\mathrm{ij}}\right)}{\ln\left(x_{j}^{\max}\right)-\ln\left(x_{j}^{\min}\right)}$$

where *x_ij_* is the reported value of criterion *j* for gyroscope *i*, $x_{j}^{\min}$ and $x_{j}^{\max}$ are the minimum and maximum values within the investigated dataset, and $n_{\mathrm{ij}}^{\log}$ is the logarithmically normalized value.

Because all reported BS and ARW values are strictly positive, the logarithmic transformation is applicable to the investigated dataset. It maps the minimum observed value to 1 and the maximum to 0 while representing equal multiplicative changes as equal differences on the normalized scale.

For Maximum Input Rate (MIR), Bandwidth (BW), Operating Temperature Range Width (TRW), and Power Consumption (PC), the original linear Min–Max normalization was retained. The resulting alternative FOGPI score was calculated as

(30) $${\mathrm{FOGPI}}_{i}^{\log}=w_{\mathrm{BS}}n_{i,\mathrm{BS}}^{\log}+w_{\mathrm{ARW}}n_{i,\mathrm{ARW}}^{\log}+\sum_{j\in \mathrm{MIR},\mathrm{BW},\mathrm{TRW},\mathrm{PC}}w_{j}n_{\mathrm{ij}}^{\mathrm{lin}}$$

where *w_j_* denotes the original criterion weight and $n_{\mathrm{ij}}^{\mathrm{lin}}$ is the corresponding linearly normalized value.

The baseline and logarithmically normalized FOGPI scores were compared using the same eleven FOGs and weighting coefficients. This comparison isolated the effect of the normalization function applied to BS and ARW. Both approaches remained dependent on the observed minimum and maximum values of the comparison dataset.

#### 2.7.2. Fixed-Reference Normalization

To investigate sample-composition dependence, an additional normalization procedure was defined using fixed lower and upper reference bounds, *L_j_* and *U_j_*, which remain unchanged when devices are added to or removed from the comparison dataset.

For cost criteria (BS, ARW, and PC), fixed linear normalization was calculated as

(31) $$n_{\mathrm{ij}}^{\mathrm{fixed}}=\frac{U_{j}-x_{\mathrm{ij}}}{U_{j}-L_{j}}$$

For benefit criteria (MIR, BW, and TRW), it was calculated as

(32) $$n_{\mathrm{ij}}^{\mathrm{fixed}}=\frac{x_{\mathrm{ij}}-L_{j}}{U_{j}-L_{j}}$$

Normalized values outside the predefined reference interval were clipped to the range [0, 1]. Here, *clip*_[0,1]_(*z*) = *min*(1,*max*(0,*z*)), ensuring that the normalized score remains within the unit interval when an observed criterion value lies outside the predefined reference bounds.

Two fixed-reference configurations were investigated: fixed linear normalization for all six criteria and fixed logarithmic normalization for BS and ARW combined with fixed linear normalization for the remaining criteria. The fixed logarithmic transformation was defined as

(33) $$n_{\mathrm{ij}}^{\mathrm{fixed},\log}={\mathrm{clip}}_{\left[0,1\right]}\left(\frac{\ln\left(U_{j}\right)-\ln\left(x_{\mathrm{ij}}\right)}{\ln\left(U_{j}\right)-\ln\left(L_{j}\right)}\right)$$

The fixed logarithmic transformation was applied only to BS and ARW because their observed values and corresponding reference bounds were strictly positive. The remaining four criteria were normalized using the fixed linear formulation.

The adopted reference intervals are presented in Table 4.

The reference bounds were selected as illustrative engineering anchors to cover a broad range of reported FOG characteristics, with published studies [65,66,67] providing general context for the variability of gyroscope specifications rather than prescribing the numerical endpoints adopted here. IEEE Std 952-2020 was used as a methodological reference for specification and testing principles rather than as a source of universal numerical normalization limits.

Both fixed-reference configurations were evaluated using the original FOGPI weighting coefficients and aggregation procedure described in Section 2.4 and Section 2.5.

To assess the influence of comparison-set composition, DSP-3000, which defines the maximum ARW value in the original dataset, was excluded. The fixed-reference FOGPI scores of the remaining ten devices were then recalculated without changing the reference bounds or weighting coefficients.

Under fixed-reference normalization, the numerical FOGPI score of an unchanged device remains invariant to the addition or removal of other alternatives, provided that its technical characteristics, reference bounds, and weighting coefficients remain unchanged. However, its ordinal rank may change when competing alternatives are added or removed. Furthermore, the resulting scores remain dependent on the selected reference intervals and transformation functions, which represent methodological assumptions rather than universally calibrated performance scales.

The resulting analyses characterize the sensitivity of FOGPI scores and rankings to normalization assumptions and comparison-set composition. The corresponding results are presented in Section 3.3.

### 2.8. Weight Sensitivity Analysis

A local one-factor-at-a-time (OFAT) sensitivity analysis was performed to examine the response of FOGPI scores and rankings to changes in individual functional-group weights. The analysis was conducted at the group level, consistent with the four-group weighting structure defined in Section 2.4.

Each group weight was varied separately by ±20% relative to its baseline value. When one group weight was changed, the remaining three group weights were proportionally rescaled while preserving their baseline relative proportions and maintaining the constraint that the sum of all group weights remained equal to unity. Within the Accuracy and Dynamic Performance groups, the corresponding group weight continued to be divided equally between their two constituent criteria.

For a modified group weight $w_{k}^{\prime }$, the remaining group weights were proportionally rescaled according to the following expression:

(34) $$w_{j}^{\prime }=w_{j}\frac{1-w_{k}^{\prime }}{1-w_{k}},\;j\neq k$$

where *w_k_* and $w_{k}^{\prime }$ are the baseline and modified weights of the varied group, respectively, while *w_j_* and $w_{j}^{\prime }$ denote the baseline and rescaled weights of each remaining group *j* ≠ *k*.

For example, varying the Accuracy weight from its baseline value of 0.50 produced values of 0.40 and 0.60. The remaining group weights were proportionally adjusted to (0.30, 0.18, 0.12) and (0.20, 0.12, 0.08), respectively. The same procedure was independently applied to the Dynamic Performance, Operating Temperature Capability, and Power Requirement groups.

For every weighting scenario, FOGPI values and the resulting ranks of all eleven FOGs were recalculated. The original technical specifications and baseline linear Min–Max normalized criterion values were held constant throughout the analysis to isolate the influence of weight variation. Rank stability relative to the baseline ranking was quantified using Spearman’s rank correlation coefficient and Kendall’s rank correlation coefficient. In addition, changes in the highest-ranked alternative were recorded to identify weight ranges associated with rank reversal.

The OFAT analysis characterizes local ranking sensitivity along four predefined weight-variation directions but does not establish stability throughout the complete admissible weighting space. To address this limitation, the analysis was complemented by mission-oriented weighting scenarios and global weight-space sampling, described in Section 2.9 and Section 2.10, respectively. The corresponding local sensitivity results are presented in Section 3.4.1.

### 2.9. Mission-Oriented Weighting Scenarios

To investigate the dependence of FOGPI rankings on application-specific engineering priorities, three illustrative mission-oriented weighting scenarios were developed: long-duration inertial navigation, UAV/tactical navigation, and compact robotic systems. These scenarios were selected to represent different relative priorities among accuracy, dynamic performance, operating-temperature capability, and power consumption.

The Analytic Hierarchy Process (AHP) was employed to derive weighting coefficients for the four functional groups: Accuracy (A), Dynamic Performance (D), Operating Temperature Capability (T), and Power Requirement (P). For each scenario, a reciprocal pairwise comparison matrix was constructed using the conventional Saaty importance scale. The comparisons represented assumed engineering priorities associated with the corresponding application rather than empirically elicited expert judgments or formally validated mission requirements.

The weighting vector was calculated as the normalized principal right eigenvector of each comparison matrix. The consistency of the pairwise comparisons was assessed using the Consistency Ratio *CR*:

(35) $$\mathrm{CR}=\frac{\mathrm{CI}}{\mathrm{RI}},\;\mathrm{CI}=\frac{\lambda_{\max}-n}{n-1}$$

where *λ_max_* is the principal eigenvalue of the pairwise comparison matrix, *n* =4 is the number of functional groups, and *RI* = 0.90 is the random consistency index for a four-criterion matrix. A consistency ratio below 0.10 was considered acceptable.

For the long-duration INS scenario, greater importance was assigned to Accuracy because gyroscope bias and random noise contribute to accumulated attitude errors during autonomous navigation. For a simplified constant-bias and white-noise model, the corresponding contributions to angular error can be approximated as

(36) $$\theta_{b}\left(t\right)\approx \mathrm{bt},\;\sigma_{\theta ,\mathrm{ARW}}\left(t\right)\approx N\sqrt{t}$$

where *b* denotes an assumed constant angular-rate bias, *N* is the angle random walk coefficient, and *t* is the integration time expressed in hours when *b* and *N* are specified in °/h and °/√h, respectively. These simplified relationships illustrate the different time dependencies of bias-induced and white-noise-induced angular errors. They provide a physical motivation for emphasizing accuracy-related characteristics in long-duration navigation but do not establish a direct equivalence between manufacturer-reported BS and constant bias, nor do they determine the numerical AHP weights.

The UAV/tactical navigation scenario assigned equal importance to Accuracy and Dynamic Performance to reflect the combined requirements of maintaining navigation accuracy and tracking rapid angular motion. In contrast, the compact robotic scenario emphasized Power Requirement and Dynamic Performance, representing the assumed priorities of energy-constrained mobile robotic platforms.

For each scenario, the AHP-derived weighting vector and consistency ratio were calculated from the corresponding pairwise comparison matrix. The adopted pairwise comparison matrices, corresponding principal eigenvectors, and consistency calculations are documented in Appendix A to support reproducibility of the scenario-based weighting procedure. The pairwise judgments were expressed using the conventional 1–9 importance scale, where 1 indicates equal importance and 9 indicates extreme importance of one criterion over another. Reciprocal values were assigned to the corresponding inverse comparisons. The resulting group weights were applied to the original linearly normalized decision matrix using the FOGPI aggregation procedure described in Section 2.5. Within the Accuracy and Dynamic Performance groups, each group weight was distributed equally between its two constituent criteria. The input specifications and normalization procedure were held constant to isolate the influence of the weighting structure. The resulting weights, consistency ratios, and application-specific rankings are presented in Section 3.4.2.

### 2.10. Global Weight-Space Sensitivity Analysis

To assess the robustness of FOGPI rankings under simultaneous variations of multiple weighting coefficients, a global weight-space sensitivity analysis was performed. Unlike the one-factor-at-a-time procedure described in Section 2.8, this analysis explored combinations of all four functional-group weights within the admissible preference space.

The admissible weighting configurations were defined by the constraints

*w_A_* + *w_D_* + *w_T_* + *w_P_* = 1,  *w_g_* ≥ 0 (37)

where *w_A_*, *w_D_*, *w_T_*, and *w_P_* denote the weights of Accuracy, Dynamic Performance, Operating Temperature Capability, and Power Requirement, respectively.

Two weight-space sampling configurations were investigated. First, a symmetric Dirichlet distribution with parameters α = (1, 1, 1, 1) was used to sample uniformly over the complete admissible weight simplex. Second, a baseline-centered Dirichlet distribution with parameters α = (25, 12.5, 7.5, 5) was used to concentrate sampled weighting configurations around the reference preference vector (0.50, 0.25, 0.15, 0.10). The concentration parameters of the second distribution were selected as an illustrative methodological assumption rather than derived from expert-elicited or empirically observed preference distributions. The baseline-centered distribution has a total concentration parameter of 50 and an expected weighting vector equal to the baseline configuration.

For each sampling configuration, 200,000 weighting vectors were independently generated. The original technical specifications and linearly normalized criterion values were held constant throughout the analysis. For each sampled weighting vector, the four functional-group scores were aggregated using the corresponding group weights, and the FOGPI scores and ranks of all eleven devices were recalculated. The empirical rank acceptability indices were subsequently estimated from the frequencies of the resulting ranking positions.

For each gyroscope, the empirical rank acceptability index was defined as the fraction of sampled weighting configurations in which the device occupied a given ranking position:

(38) $$b_{i}\left(r\right)=\frac{1}{N}\sum_{k=1}^{N}I\left(R_{i}^{\left(k\right)}=r\right)$$

where *N* is the number of sampled weighting configurations, $R_{i}^{\left(k\right)}$ is the rank assigned to gyroscope *i* under weighting configuration *k*, and *I*(⋅) is the indicator function.

The first-place acceptability index *b_i_*(1) and cumulative top-three acceptability, defined as $b_{i}^{\mathrm{top}3}=\sum_{r=1}^{3}b_{i}\left(r\right)$, were used to characterize ranking stability.

The resulting rank acceptability indices represent conditional frequencies under the specified weight-space sampling distributions and should not be interpreted as empirically established probabilities of engineering preferences. The analysis was intended to identify preference regions supporting different ranking outcomes and to assess whether the baseline ranking remains stable under simultaneous changes in group weights. The corresponding results are presented in Section 3.4.3.

### 2.11. Intercriterion Dependence and Correlation-Aware Weighting

To investigate statistical dependencies among the six evaluation criteria and their potential influence on the FOGPI weighting structure, a two-stage analysis was performed. First, pairwise Spearman rank correlations were calculated to identify monotonic associations and potential redundancy among the technical characteristics. Second, the Criteria Importance Through Intercriteria Correlation (CRITIC) method was applied to examine how criterion variability and intercriterion correlations affect the resulting weights and FOGPI rankings.

For the correlation analysis, the original observations of Bias Stability (BS), Angle Random Walk (ARW), Maximum Input Rate (MIR), Bandwidth (BW), Operating Temperature Range Width (TRW), and Power Consumption (PC) were converted into ranks across the eleven investigated FOGs. Because several criteria contained repeated values, average ranks were assigned to tied observations. Spearman’s rank correlation coefficient was calculated as the Pearson correlation coefficient between the rank-transformed variables:

(39) $$\rho_{s}=\frac{\sum_{i=1}^{m}(R_{\mathrm{Xi}}-{\bar{R}}_{X})(R_{\mathrm{Yi}}-{\bar{R}}_{Y})}{\sqrt{\sum_{i=1}^{m}{\left(R_{\mathrm{Xi}}-{\bar{R}}_{X}\right)}^{2}\sum_{i=1}^{m}{\left(R_{\mathrm{Yi}}-{\bar{R}}_{Y}\right)}^{2}}}$$

where *R_Xi_* and *R_Yi_* are the ranks of the *i*-th FOG according to criteria *X* and *Y*, respectively; ${\bar{R}}_{X}$ and ${\bar{R}}_{Y}$ are the corresponding mean ranks; and *m* = 11 is the number of investigated devices.

The correlation analysis was used as a diagnostic assessment rather than a criterion-elimination procedure. Strong statistical associations do not necessarily imply physical equivalence, because correlated gyroscope characteristics may describe different aspects of sensor performance. Accordingly, all six criteria were retained in the evaluation framework.

To assess the influence of intercriterion dependencies on the weighting structure, an additional analysis was performed using the CRITIC method. Unlike the baseline engineering preference weights, CRITIC derives criterion weights from the statistical characteristics of the decision matrix by considering both the variability of normalized criterion values and their correlations.

The analysis was performed using the original eleven-device decision matrix and the linear Min–Max normalization procedure described in Section 2.3. All six criteria were transformed into benefit-oriented normalized values before calculating the CRITIC information content, ensuring that higher normalized values consistently represented more favorable technical characteristics. For each criterion *j*, the CRITIC information content was calculated as:

(40) $$C_{j}=\sigma_{j}\sum_{k=1}^{m}\left(1-r_{\mathrm{jk}}\right)$$

where *σ_j_* is the standard deviation of the normalized values of criterion *j*, *r_jk_* is the Pearson correlation coefficient between normalized criteria *j* and *k*, and *m* = 6 is the number of evaluation criteria.

The resulting CRITIC weight was calculated as:

(41) $$w_{j}^{\mathrm{CRITIC}}=\frac{C_{j}}{\sum_{k=1}^{m}C_{k}}$$

The conventional CRITIC formulation employs Pearson correlation coefficients to quantify linear relationships between normalized criteria, whereas the preceding Spearman analysis examines monotonic associations in the original technical characteristics. The two analyses therefore serve different but complementary purposes.

The CRITIC-derived weights were subsequently applied to the same weighted additive aggregation procedure used in the baseline FOGPI model. The original technical specifications and normalization procedure were held constant, allowing differences in the resulting rankings to be attributed to the weighting strategy.

The CRITIC weights were interpreted as data-dependent statistical weights rather than physically validated priorities for inertial-navigation applications. Accordingly, the analysis was intended to assess weighting-method sensitivity and the influence of intercriterion dependence, not to establish CRITIC as a universally superior weighting approach. The corresponding Spearman correlation and CRITIC weighting results are presented in Section 3.5.

### 2.12. Ranking Robustness Under Dataset Changes and Input Uncertainty

To investigate the robustness of FOGPI scores and rankings, two complementary analyses were conducted. The first examined sensitivity to the composition of the comparison dataset using a leave-one-out (LOO) procedure. The second assessed the influence of hypothetical uncertainty in manufacturer-reported technical specifications using Monte Carlo simulation. These analyses address different sources of ranking variability and were therefore performed separately.

#### 2.12.1. Leave-One-Out Analysis

Because the baseline Min–Max normalization determines criterion bounds from the observed minimum and maximum values, FOGPI scores may depend on which devices are included in the comparison dataset. To quantify this dependence, each of the eleven FOGs was removed individually, generating eleven reduced datasets containing ten devices each.

For every reduced dataset, the normalization bounds were recalculated, the six criteria were renormalized, and FOGPI scores and ranks were obtained using the original baseline weighting configuration. The ordering of the remaining devices was compared with their corresponding baseline ordering using Spearman’s rank correlation coefficient and Kendall’s rank correlation coefficient. Changes in the highest-ranked device and individual rank reversals were also recorded.

The LOO analysis was intended to identify alternatives whose extreme criterion values exert a substantial influence on the normalization bounds and to evaluate whether the relative ordering of unchanged devices remains stable when the comparison set changes.

#### 2.12.2. Monte Carlo Uncertainty Analysis

To examine sensitivity to uncertainty in the input specifications, a scenario-based Monte Carlo simulation was performed. Because consistent measurement uncertainties, device-to-device variability estimates, and probability distributions were unavailable for all eleven FOGs, the simulation employed hypothetical relative perturbations rather than experimentally established uncertainty models.

Three perturbation levels were considered: ±5%, ±10%, and ±20%. For each gyroscope *i*, criterion *j*, and iteration *k*, the perturbed input value was calculated as

(42) $$x_{\mathrm{ij}}^{\left(k\right)}=x_{\mathrm{ij}}^{\left(0\right)}\left(1+\delta_{\mathrm{ij}}^{\left(k\right)}\right)$$

where $x_{\mathrm{ij}}^{\left(0\right)}$ is the original manufacturer-reported specification and

(43) $$\delta_{\mathrm{ij}}^{\left(k\right)}∼U\left(-u,u\right),u\in \{0.05,0.10,0.20\}$$

Perturbations were sampled independently for each device and criterion. This assumption was adopted for exploratory analysis and does not imply that actual measurement or manufacturing uncertainties are statistically independent.

For each of the three perturbation levels, 100,000 Monte Carlo realizations were evaluated under both linear and logarithmic normalization, yielding six sets of 100,000 FOGPI score and ranking evaluations.

Two normalization configurations were investigated: linear Min–Max normalization for all six criteria and logarithmic normalization for BS and ARW combined with linear normalization for MIR, BW, TRW, and PC, as described in Section 2.7.

The resulting scores were ranked in descending order for each iteration. In the event of exactly equal FOGPI scores, tied alternatives were assigned the same competition rank. Accordingly, alternatives sharing the highest score were jointly counted as first-ranked devices. No arbitrary ordering was introduced to resolve ties. The empirical rank acceptability of gyroscope *i* at position *r* was estimated as

(44) $$P_{i}\left(r\right)=\frac{1}{N}\sum_{k=1}^{N}I\left(R_{i}^{\left(k\right)}=r\right)$$

where *N* = 100,000, $R_{i}^{\left(k\right)}$ is the rank assigned to gyroscope *i* in iteration *k*, and *I*(⋅) is the indicator function, equal to 1 when the specified condition is satisfied and 0 otherwise.

The simulation results were summarized using the mean FOGPI score, the 2.5th and 97.5th percentiles of the simulated score distribution, the empirical frequency of occupying first place, and the cumulative frequency of appearing among the three highest-ranked devices.

The resulting rank frequencies and score intervals are conditional on the assumed perturbation distributions. They should not be interpreted as experimentally established probabilities of device superiority or formal metrological confidence intervals. The purpose of this analysis was to determine how hypothetical variations in the reported specifications affect the stability of comparative rankings. The adopted perturbation model does not resolve missing or ambiguously documented manufacturer specifications and should not be interpreted as a substitute for independent source verification.

The corresponding LOO and Monte Carlo results are presented in Section 3.6.1 and Section 3.6.2 respectively.

### 2.13. Application-Specific Admissibility and Temperature Screening

The weighted additive structure of FOGPI is compensatory, meaning that relatively weak performance in one criterion may be partially offset by favorable values in other criteria. Although this property supports comparative evaluation of heterogeneous performance profiles, it may be inappropriate when a particular application imposes mandatory technical requirements.

To distinguish preference-based ranking from requirement-based selection, a two-stage decision procedure was adopted. In the first stage, each candidate FOG was evaluated against application-specific admissibility constraints. For cost criteria, admissibility was defined by the following inequality:

(45) $$x_{\mathrm{ij}}\leq U_{j}^{\mathrm{req}},\;j\in J_{\mathrm{cost}}$$

For benefit criteria, admissibility was defined as:

(46) $$x_{\mathrm{ij}}\geq L_{j}^{\mathrm{req}},\;j\in J_{\mathrm{benefit}}$$

where *x_ij_* is the reported value of criterion *j* for gyroscope *i*, $U_{j}^{\mathrm{req}}$ is the maximum admissible value of a cost criterion, and $L_{j}^{\mathrm{req}}$ is the minimum required value of a benefit criterion. A device was considered admissible only if all mandatory requirements were satisfied.

In the second stage, the admissible alternatives were ranked using their original baseline FOGPI scores calculated from the complete eleven-device dataset. The normalization bounds and weighting coefficients were not recalculated after screening, thereby preventing changes in the scores of unchanged alternatives solely due to the removal of inadmissible devices. Under this procedure, favorable performance in secondary criteria cannot compensate for failure to satisfy mandatory technical requirements.

To demonstrate the application of this procedure, two illustrative engineering scenarios were defined: a high-accuracy inertial navigation system (INS) and a compact unmanned aerial vehicle (UAV).

For the INS scenario, the mandatory requirements were BS ≤ 0.01°/h, ARW ≤ 0.002°/√h, MIR ≥ 300°/s, BW ≥ 100 Hz, PC ≤ 6 W, and an operating-temperature interval covering −40 to +65 °C. This scenario represents a hypothetical navigation application emphasizing accuracy-related requirements.

For the compact UAV scenario, the requirements were BS ≤ 1.0°/h, ARW ≤ 0.04°/√h, MIR ≥ 300°/s, BW ≥ 300 Hz, PC ≤ 2 W, and an operating-temperature interval covering −20 to +60 °C. This scenario emphasizes power efficiency and sufficient dynamic capability while adopting less restrictive accuracy requirements.

The specified thresholds represent illustrative engineering assumptions rather than standardized qualification limits or formally documented mission requirements. The operating-temperature requirement was evaluated using the separately reported minimum and maximum operating temperatures rather than Operating Temperature Range Width (TRW) alone.

To further investigate the limitations of TRW as a standalone measure of thermal capability, an additional temperature-specific admissibility assessment was performed. For each investigated gyroscope, the manufacturer-reported minimum and maximum operating temperatures, *T_min_*_,*i*_ and *T_max_*_,*i*_, were retained separately, with

*TRW_i_* = *T_max_*_,*i*_ − *T_min_*_,*i*_ (47)

Thermal admissibility for a specified application environment was defined as

*E_i_* = *I*(*T_min_*_,*i*_ ≤ *T_req_*_,*min*_) ⋅ *I*(*T_max_*_,*i*_ ≥ *T_req_*_,*max*_) (48)

where *I*(⋅) is the indicator function, equal to 1 when the corresponding condition is satisfied and 0 otherwise. The indicator *E_i_* = 1 denotes that both required temperature limits are covered by the manufacturer’s specified operating interval.

Two additional illustrative thermal scenarios were examined: extended cold operation (−45 to +65 °C) and extended upper-temperature operation (−40 to +75 °C). These scenarios were selected to demonstrate that devices with identical TRW values may exhibit different thermal admissibility because their operating intervals have different absolute limits.

The thermal assessment was conducted as a mandatory screening procedure rather than as an additional weighted criterion. Devices failing either temperature requirement were considered inadmissible for the corresponding scenario. The original FOGPI scores and baseline normalization bounds were retained for admissible alternatives.

The assessment was based on manufacturer-reported operating-temperature limits and did not establish that BS, ARW, scale-factor characteristics, or other accuracy-related parameters remain constant throughout the specified temperature interval. The investigated scenarios therefore illustrate the application of mandatory technical constraints rather than certify device suitability for specific operational platforms.

The results of the temperature-specific assessment and the application-specific two-stage selection procedure are presented in Section 3.8.1 and Section 3.8.2, respectively.

## 3. Results

This section presents the results of the proposed six-criterion comparative evaluation framework applied to eleven fiber-optic gyroscopes. The analysis begins with the examination of reported technical characteristics and specification comparability, followed by baseline FOGPI calculation. The sensitivity of the resulting scores and rankings is then investigated with respect to normalization procedures, weighting configurations, intercriterion dependencies, comparison-set composition, and hypothetical input-specification uncertainty. The results are subsequently compared with TOPSIS and VIKOR rankings, and application-specific admissibility screening is used to demonstrate the distinction between aggregate preference-based ranking and mandatory engineering requirements.

### 3.1. Dataset Characteristics and Specification Comparability

The initial analysis examined the reported technical characteristics and specification comparability of the eleven investigated fiber-optic gyroscopes. Particular attention was given to the distribution of the accuracy-related criteria, Bias Stability (BS) and Angle Random Walk (ARW), and to differences in the statistical definitions and operating conditions associated with manufacturer-reported specifications.

#### 3.1.1. Reported Accuracy Characteristics

**Figure 1:**
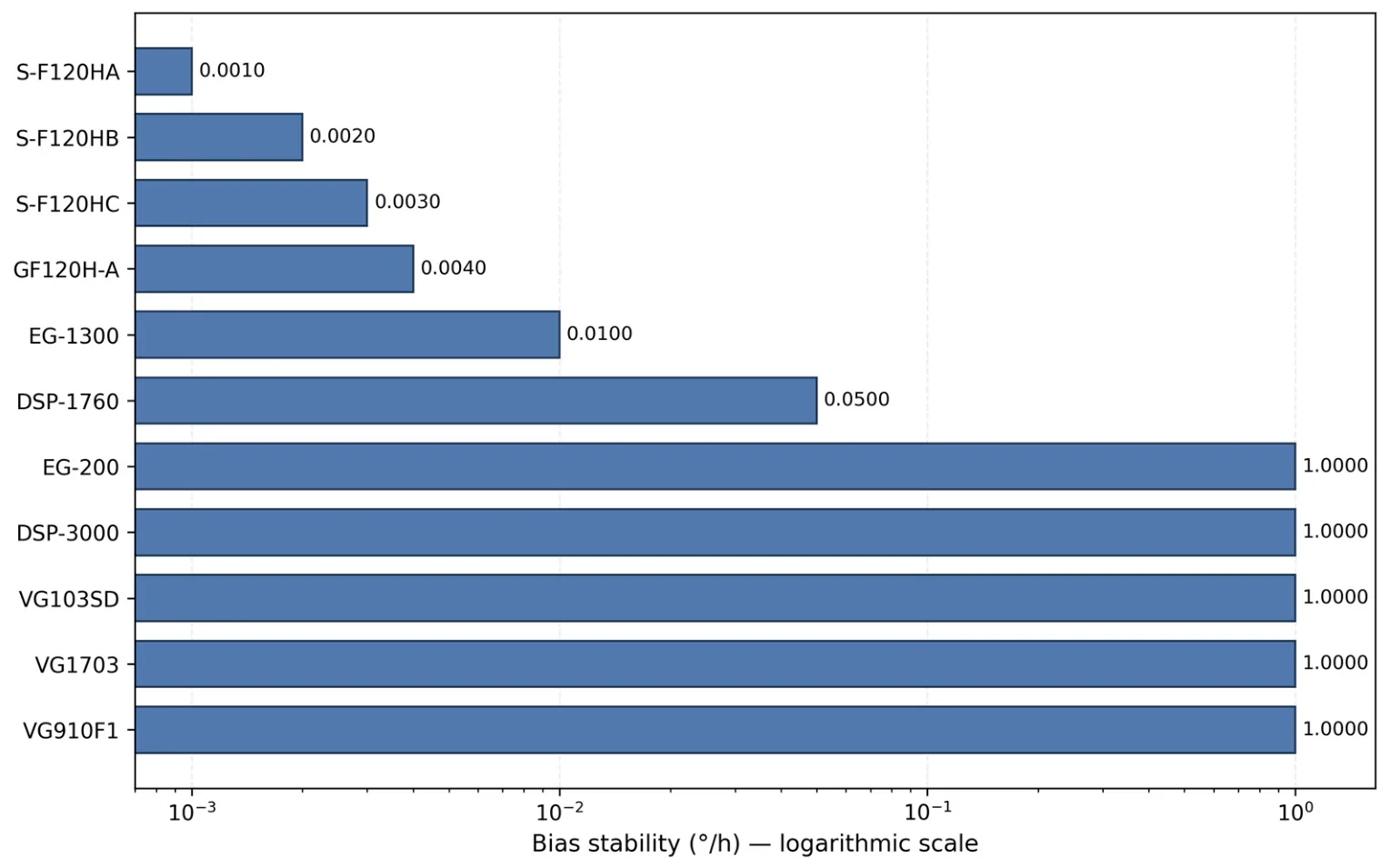
Reported Bias Stability values of the eleven investigated FOGs (logarithmic horizontal axis).

The manufacturer-reported bias-related stability values varied substantially across the investigated devices, as illustrated in Figure 1. These values were treated under the common BS designation for comparative purposes, although their statistical definitions and measurement conditions were not necessarily equivalent. The S-F120HA, S-F120HB, and S-F120HC models exhibited the lowest reported values, ranging from 0.001 to 0.003°/h. GF120H-A and EG-1300 also had comparatively low values of 0.004 and 0.010°/h, respectively. DSP-1760 had a reported BS value of 0.050°/h, whereas EG-200, DSP-3000, VG103SD, VG1703, and VG910F1 each had a reported value of 1.000°/h.

**Figure 2:**
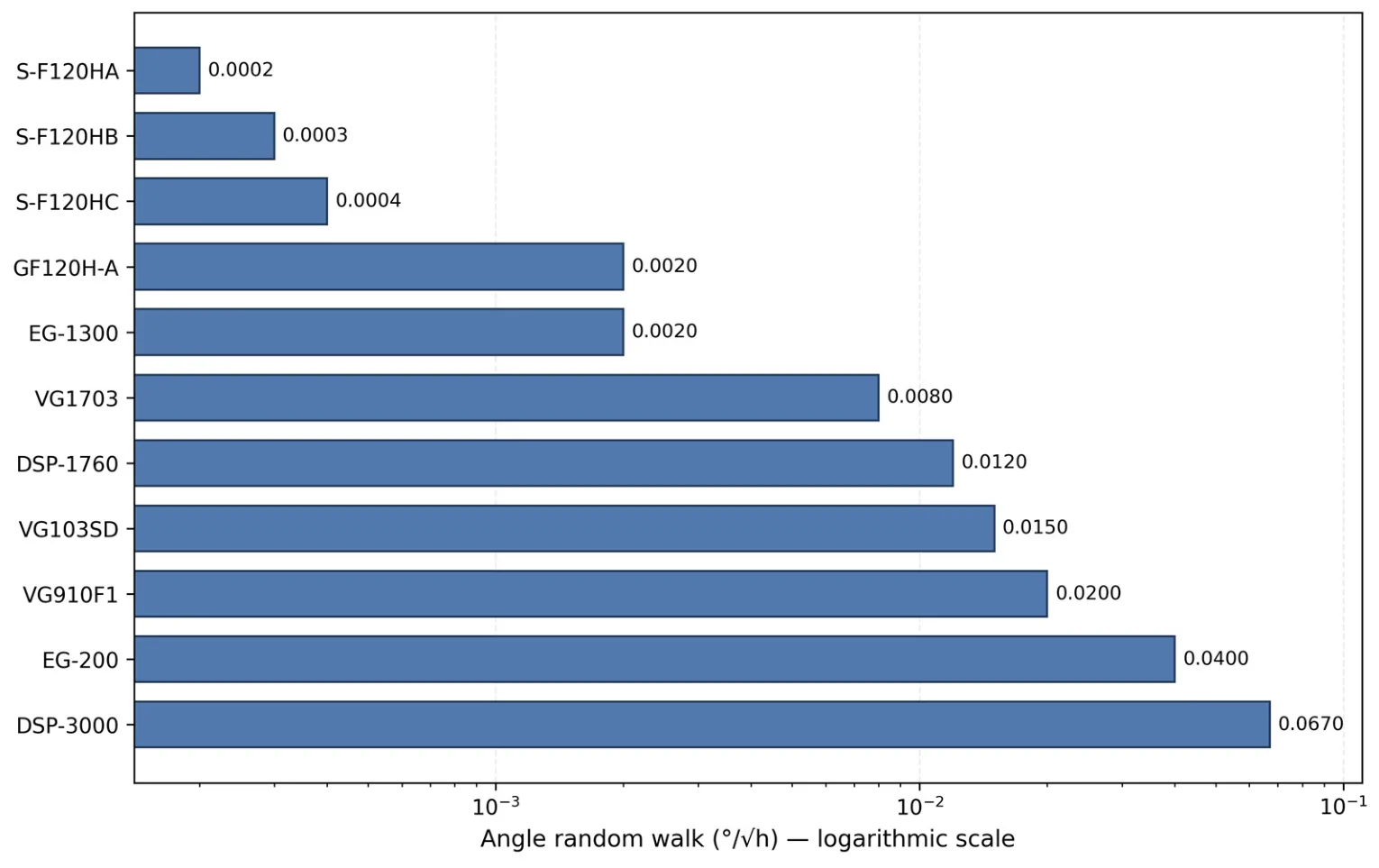
Reported Angle Random Walk values of the eleven investigated FOGs (logarithmic horizontal axis).

The reported ARW values also varied considerably, as shown in Figure 2. The S-F120H series exhibited the lowest values, ranging from 0.0002 to 0.0004°/√h. GF120H-A and EG-1300 each had a reported ARW value of 0.002°/√h, while DSP-1760 had a value of 0.012°/√h. Among the remaining devices, the reported values ranged from 0.008 to 0.067°/√h, with DSP-3000 exhibiting the highest ARW value in the investigated dataset.

These results demonstrate substantial numerical differences in the reported accuracy-related characteristics. However, the observed differences should not be interpreted as fully standardized experimental comparisons because the underlying specification definitions and measurement conditions are not identical across the investigated devices.

#### 3.1.2. Specification Traceability and Metrological Comparability

The document-based assessment identified differences in the statistical definitions, measurement conditions, and operating configurations used to report the technical characteristics of the investigated FOGs. The assessment was guided by the specification and testing principles of IEEE Std 952-2020 [37], without assuming that the devices had been evaluated or certified under a common standardized testing procedure.

The available specification definitions and reporting conditions are summarized in Table 5.

The assessment revealed differences in the definitions and measurement conditions associated with bias-related characteristics. For example, the S-F120H series specifies BS using a 100 s averaging interval and a 1σ criterion, whereas EG-1300 and DSP-3000 report bias-related characteristics at 25 °C. The MostaTech documentation uses RMS terminology, and the specifications for VG1703 and VG910F1 additionally refer to Allan-variance-based characteristics. These differences limit direct metrological comparability between the reported BS values.

Differences were also identified in the reporting of bandwidth and power consumption. DSP-1760 specifies typical and maximum single-axis power consumption of 3 W and 6 W, respectively, whereas the DSP-3000 digital configuration reports 1.25 W typical and 3 W maximum. Furthermore, the reported bandwidth characteristics are associated with different operating configurations and measurement definitions. Some values represent specified lower limits, while others correspond to −3 dB bandwidth measurements.

For GF120H-A, the available model-specific documentation reports ARW, MIR, and operating-temperature specifications consistent with the values used in the decision matrix. It also provides two zero-bias stability limits under different averaging conditions, of which the 0.004°/h value was selected for the comparative analysis. However, the bandwidth and power-consumption values used in the decision matrix could not be independently verified for the exact model configuration. These values were retained as provisional inputs, and the corresponding limitations in specification traceability were documented.

The assessment also showed that several numerical specifications represent manufacturer-defined limits rather than independently measured performance values. Consequently, the use of these characteristics as deterministic inputs in the decision matrix introduces limitations associated with differences in reporting conventions and operating conditions.

Overall, the investigated dataset provides a basis for comparing reported FOG characteristics but does not establish complete metrological equivalence among the eleven devices. The resulting FOGPI scores should therefore be interpreted as conditional on the selected technical specifications, their definitions, and their operating configurations rather than as experimentally validated measures of intrinsic gyroscope performance.

### 3.2. Baseline FOGPI Evaluation

The baseline FOGPI evaluation was performed using the eleven investigated fiber-optic gyroscopes and the six technical criteria defined in Section 2.2. The original specifications were transformed using linear Min–Max normalization, with lower values preferred for BS, ARW, and PC and higher values preferred for MIR, BW, and TRW. The resulting normalized decision matrix is presented in Table 6.

All normalized criteria were expressed on a common dimensionless scale from 0 to 1, where higher values indicate more favorable characteristics within the investigated comparison dataset. The S-F120H series exhibited the highest normalized accuracy-related scores, whereas other devices demonstrated more favorable values for selected dynamic, operating-temperature, or power-related criteria.

**Figure 3:**
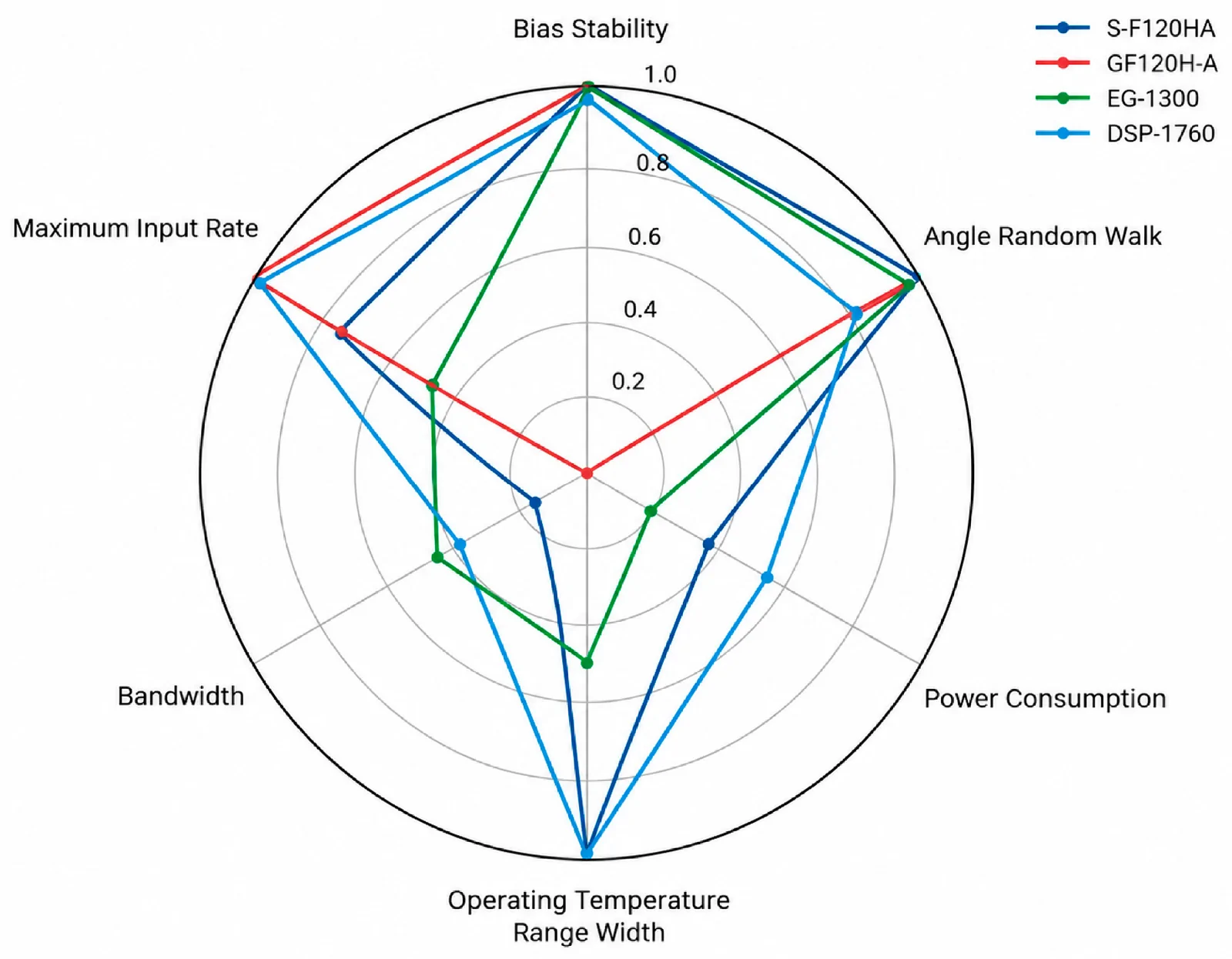
Normalized performance profiles of four representative FOG models under baseline linear Min–Max normalization.

Figure 3 illustrates the normalized performance profiles of four representative FOG models. S-F120HA exhibits the most favorable BS and ARW values but a comparatively low normalized BW score. GF120H-A combines favorable accuracy-related characteristics with the highest normalized MIR value, although its BW, TRW, and PC scores are the lowest in the investigated dataset. EG-1300 exhibits favorable accuracy-related values and intermediate scores for the remaining criteria. DSP-1760 combines relatively favorable accuracy-related and dynamic characteristics with the maximum normalized TRW value and a higher normalized PC score than the other three illustrated devices.

These profiles demonstrate that the investigated devices exhibit different combinations of strengths and limitations. None of the four illustrated models simultaneously achieves the most favorable normalized value across all six criteria.

For the baseline evaluation, the normalized characteristics were combined into four functional-group scores: Accuracy, Dynamic Performance, Operating Temperature Capability, and Power Requirement. The corresponding group weights were 0.50, 0.25, 0.15, and 0.10, respectively. The resulting group scores, FOGPI values, and ranking positions are presented in Table 7.

Under the baseline weighting configuration, DSP-1760 achieved the highest reported FOGPI score of 0.8170. The S-F120HA, S-F120HB, and S-F120HC models occupied the second, third, and fourth positions, with scores of 0.7915, 0.7908, and 0.7902, respectively. EG-1300 ranked fifth (0.6972), followed by GF120H-A in sixth position (0.6175). VG910F1, VG103SD, and VG1703 occupied positions seven to nine, whereas EG-200 and DSP-3000 occupied the final two positions.

**Figure 4:**
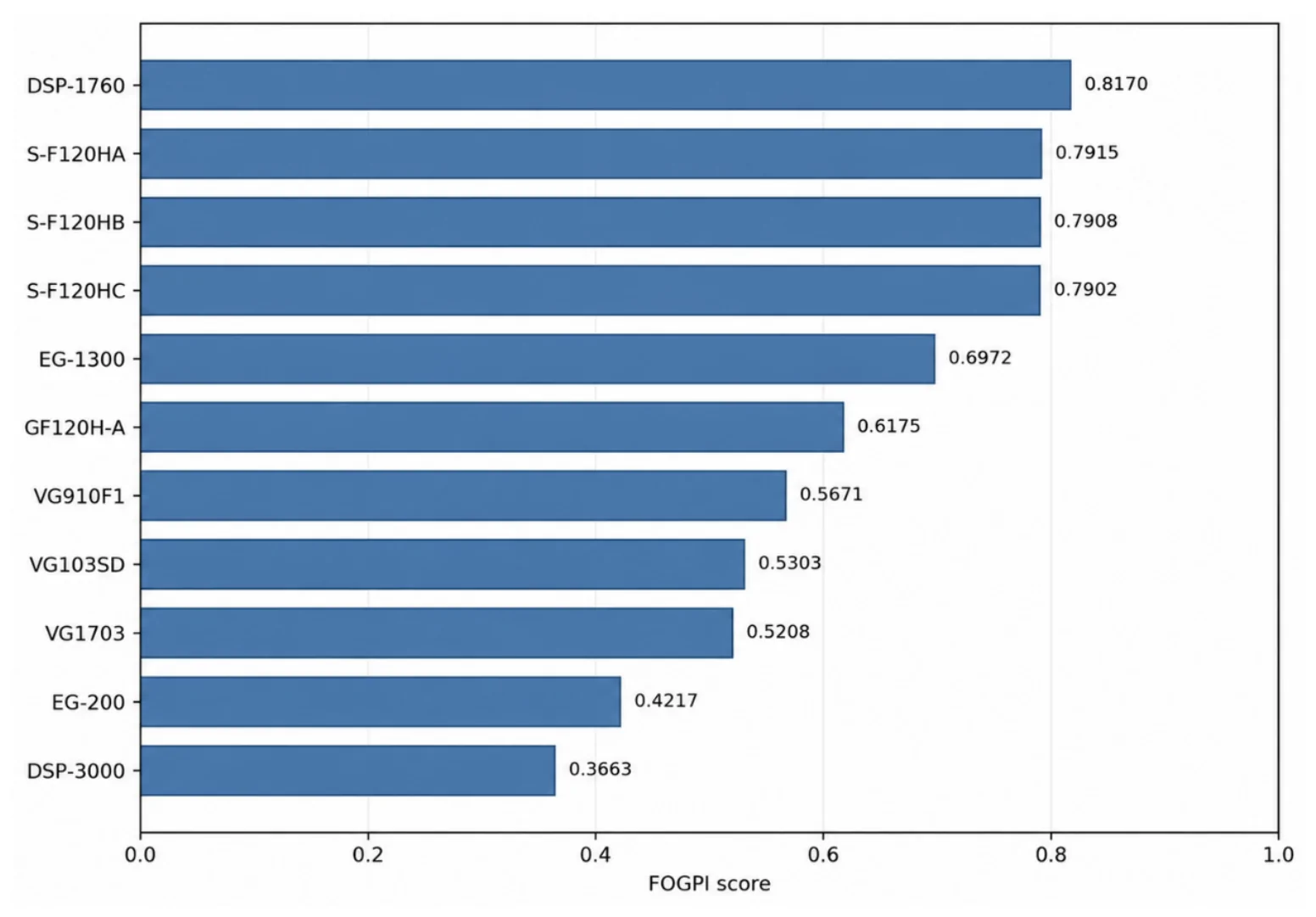
Baseline FOGPI scores and ranking of the eleven investigated FOGs under linear Min–Max normalization and the reference weighting configuration.

The resulting baseline ranking is illustrated in Figure 4.

The highest aggregate score of DSP-1760 reflects its combination of relatively favorable accuracy-related characteristics, high maximum input rate, broad operating-temperature interval, and moderate power consumption. In contrast, the S-F120H series benefits primarily from its highly favorable BS and ARW specifications, while receiving lower normalized scores for bandwidth and power consumption.

The baseline results demonstrate that the relative ordering of the investigated FOGs depends on the combined contribution of multiple technical characteristics rather than on a single performance parameter. However, the resulting scores and ranks are conditional on the selected comparison dataset, normalization procedure, and weighting configuration. They should therefore be interpreted as specification-based comparative results rather than evidence of universal device superiority.

The sensitivity of these results to alternative normalization procedures, weighting strategies, and input-data variations is examined in the following sections.

### 3.3. Normalization Sensitivity and Fixed-Reference Results

To examine the dependence of FOGPI scores and rankings on the normalization procedure, two complementary analyses were conducted. First, the baseline linear Min–Max normalization was compared with logarithmic normalization of the accuracy-related criteria, Bias Stability (BS) and Angle Random Walk (ARW). Second, fixed-reference normalization was evaluated to investigate the influence of predefined normalization bounds on FOGPI scores and their dependence on the composition of the comparison dataset.

#### 3.3.1. Influence of Logarithmic Normalization

The baseline FOGPI scores were compared with those obtained using logarithmic normalization for BS and ARW while retaining linear Min–Max normalization for Maximum Input Rate (MIR), Bandwidth (BW), Operating Temperature Range Width (TRW), and Power Consumption (PC). The same eleven FOGs and baseline weighting coefficients were used in both configurations. The resulting scores and rankings are presented in Table 8.

The results indicate that the normalization function substantially influences the ordering of the investigated devices. Under baseline linear Min–Max normalization, DSP-1760 achieves the highest FOGPI score (0.816965), followed by S-F120HA (0.791469), S-F120HB (0.790844), and S-F120HC (0.790220). When logarithmic normalization is applied to BS and ARW, S-F120HA becomes the highest-ranked device (0.791469), followed by S-F120HB (0.748948) and S-F120HC (0.721904). DSP-1760 decreases to fourth position (0.555757).

This rank reversal is associated with the different representation of relative differences in accuracy-related characteristics. For example, the reported BS values of S-F120HA and DSP-1760 are 0.001 and 0.050°/h, respectively, corresponding to a fiftyfold difference. Under linear Min–Max normalization, their normalized BS values are 1.000000 and 0.950951, whereas logarithmic normalization produces values of 1.000000 and 0.433677.

A similar effect is observed for ARW. The normalized ARW value of DSP-1760 decreases from 0.823353 under linear normalization to 0.295794 under logarithmic normalization, while S-F120HA retains the maximum normalized value of 1.000000. Because BS and ARW jointly account for 50% of the total baseline weight, these differences substantially influence the aggregate scores.

**Figure 5:**
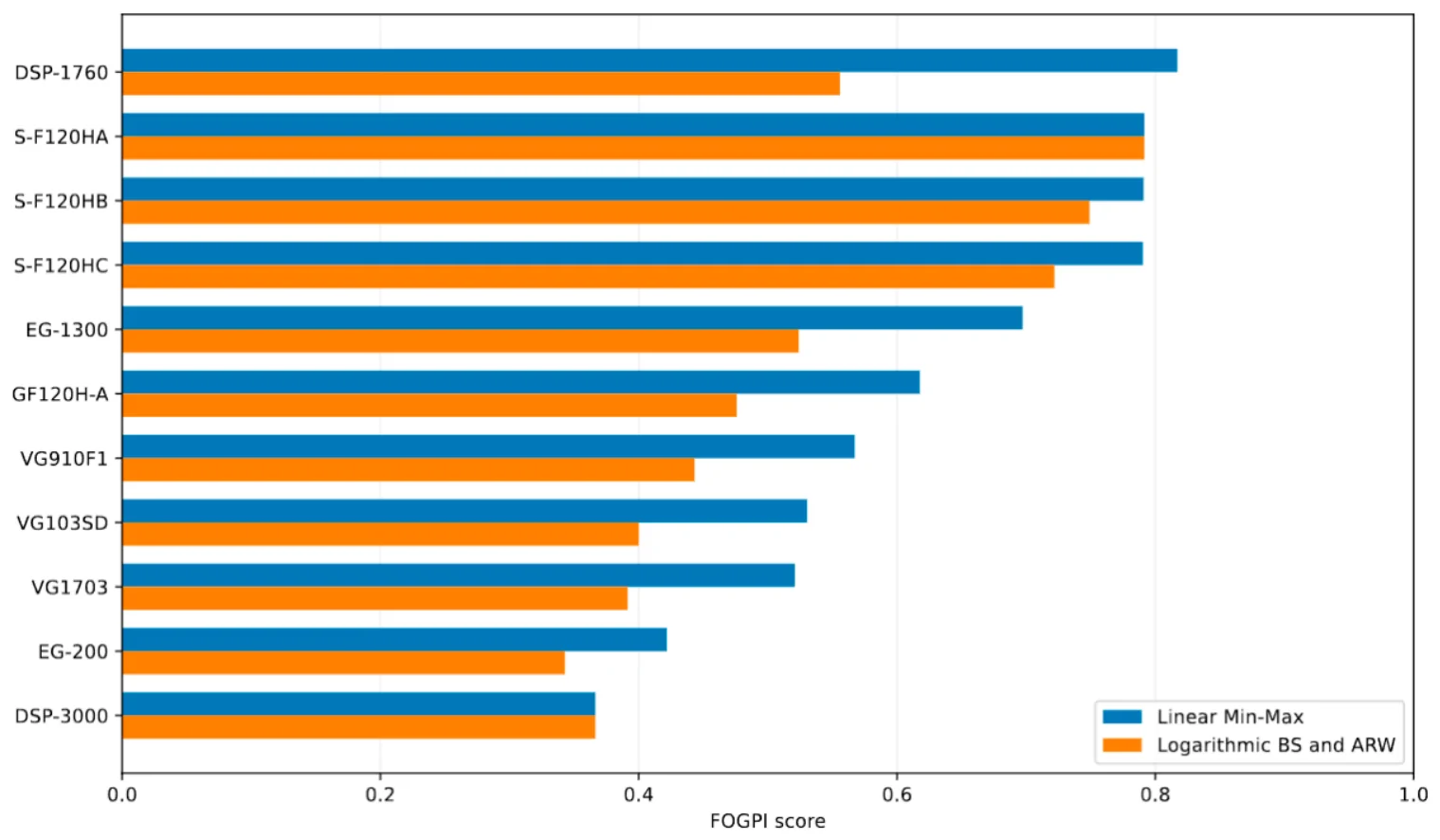
Comparison of FOGPI scores obtained using baseline linear Min–Max normalization and logarithmic normalization of BS and ARW.

The resulting changes in FOGPI scores are illustrated in Figure 5.

The results demonstrate that the highest-ranked FOG depends on the normalization function even when the technical specifications, criterion weights, and aggregation procedure remain unchanged. Logarithmic normalization emphasizes multiplicative differences in accuracy-related characteristics, whereas linear Min–Max normalization preserves relative arithmetic spacing within the investigated dataset.

#### 3.3.2. Influence of Fixed-Reference Normalization

The second analysis examined two fixed-reference configurations: fixed linear normalization for all six criteria and fixed logarithmic normalization for BS and ARW combined with fixed linear normalization for the remaining criteria. The reference bounds defined in Section 2.7 were held constant throughout the calculations.

The resulting scores were compared with the original sample-dependent FOGPI values, as presented in Table 9.

The fixed-reference configurations produced different score distributions and ranking outcomes. Under the original sample-dependent normalization, DSP-1760 achieved the highest score (0.816965). Under fixed linear normalization, VG910F1 became the highest-ranked device (0.805202), followed by VG1703 (0.801482) and DSP-1760 (0.770223). In contrast, fixed logarithmic normalization favored S-F120HA (0.674914), followed by S-F120HB (0.645188) and S-F120HC (0.625972).

To examine score dependence on the comparison dataset, DSP-3000 was removed and the fixed-reference scores of the remaining ten devices were recalculated using the same reference bounds and weighting coefficients. All remaining devices retained exactly the same fixed-reference scores within numerical precision. The maximum absolute score difference was zero for both fixed-reference configurations.

This result confirms the mathematical invariance of fixed-reference scores to changes in the comparison set when the technical characteristics, reference bounds, and weighting coefficients remain unchanged. However, the ordinal rank of a device may still change when competing alternatives are added or removed.

The results also demonstrate that eliminating sample-dependent normalization does not eliminate sensitivity to the choice of reference bounds or transformation functions. The adopted fixed bounds represent illustrative engineering anchors rather than universally standardized limits. Therefore, the resulting scores should be interpreted as conditional on the selected reference scales.

Overall, the two analyses show that normalization affects both the relative emphasis assigned to technical differences and the dependence of numerical scores on the comparison dataset. Consequently, normalization should be regarded as an explicit methodological choice in FOGPI-based comparative evaluation rather than as a neutral preprocessing operation.

### 3.4. Weighting Sensitivity and Mission-Oriented Results

The influence of weighting assumptions on FOGPI scores and rankings was examined through three complementary analyses: local one-factor-at-a-time (OFAT) sensitivity, mission-oriented weighting scenarios based on the Analytic Hierarchy Process (AHP), and global weight-space sampling. These analyses were conducted using the same eleven-device decision matrix and baseline linear Min–Max normalization, allowing the effects of different weighting configurations to be examined independently of changes in the technical specifications.

#### 3.4.1. Local Weight Sensitivity

The local sensitivity analysis examined the effect of independently varying each functional-group weight by ±20% while proportionally rescaling the remaining weights. The resulting weighting configurations, highest-ranked devices, and rank correlations relative to the baseline ranking are summarized in Table 10.

The ranking remained unchanged under the investigated variations in Dynamic Performance, Operating Temperature Capability, and Power Requirement weights. In all six corresponding scenarios, Spearman’s rank correlation coefficient and Kendall’s rank correlation coefficient were equal to 1.000.

The Accuracy group produced greater ranking sensitivity. Reducing its weight from 0.50 to 0.40 retained DSP-1760 as the highest-ranked device, with Spearman’s ρ = 0.973 and Kendall’s τ = 0.927 relative to the baseline ranking. Increasing the Accuracy weight to 0.60 changed the highest-ranked device to S-F120HA, with ρ = 0.936 and τ = 0.855.

**Figure 6:**
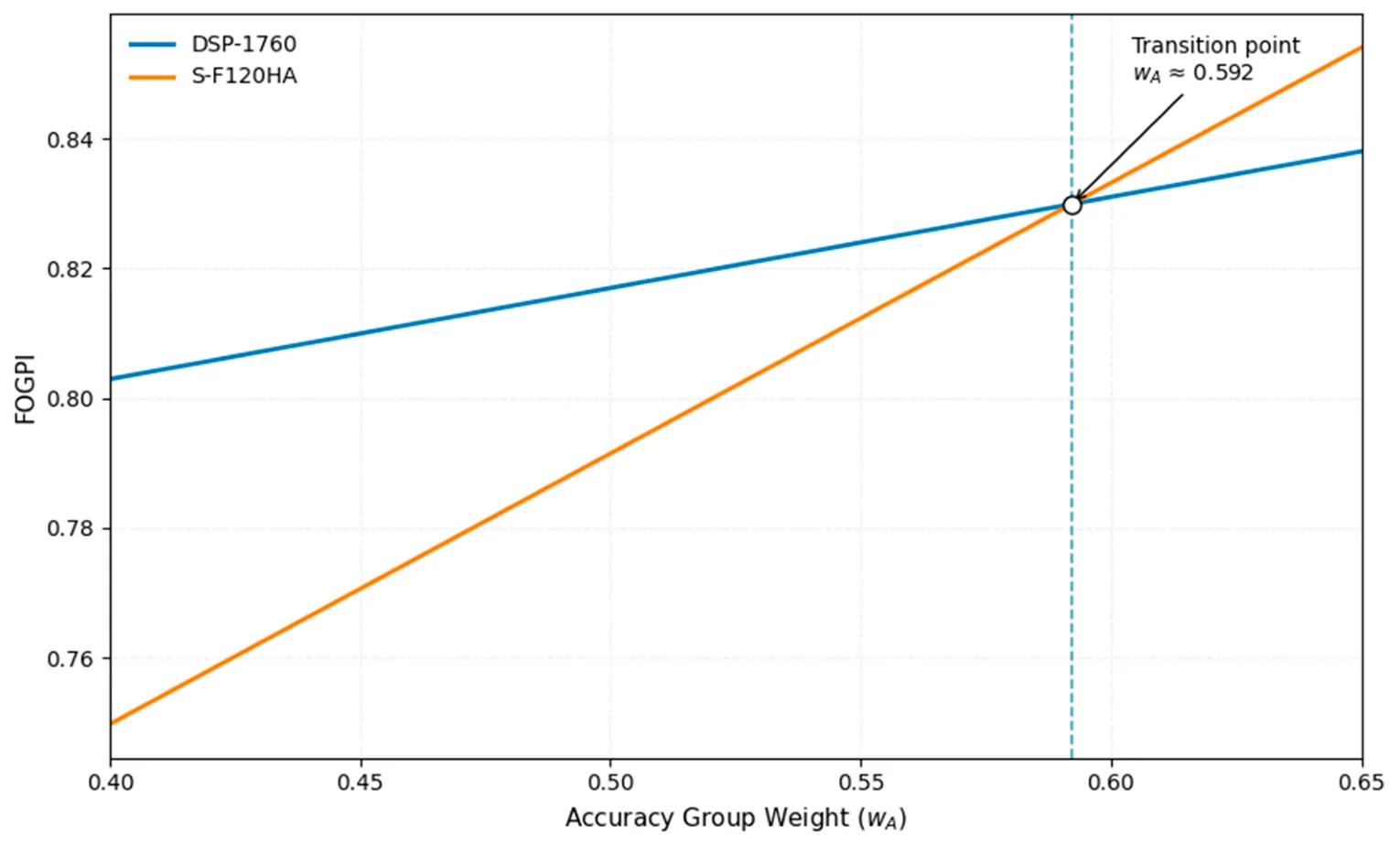
Sensitivity of the FOGPI scores of DSP-1760 and S-F120HA to changes in the Accuracy group weight. The vertical dashed line indicates the transition point *w_A_* ≈ 0.592, at which the two devices have equal FOGPI scores.

A more detailed examination identified a transition between DSP-1760 and S-F120HA at an Accuracy group weight of approximately 0.592, with the remaining group weights proportionally rescaled according to their baseline proportions. Below this threshold, DSP-1760 retained the highest FOGPI score, whereas S-F120HA became the highest-ranked alternative above it. The sensitivity of FOGPI scores to changes in the Accuracy group weight is illustrated in Figure 6.

These results show that the baseline leader is sensitive to the importance assigned to Accuracy, even within the investigated local variation range. However, the OFAT analysis examines only four predefined directions in the weighting space and cannot establish ranking stability under simultaneous changes in multiple weights.

#### 3.4.2. Mission-Oriented Weighting Scenarios

To examine the influence of application-specific engineering priorities on FOGPI rankings, three illustrative mission-oriented weighting scenarios were evaluated using the Analytic Hierarchy Process (AHP): long-duration inertial navigation, UAV/tactical navigation, and compact robotic systems. The pairwise comparison matrices represented assumed engineering preferences among Accuracy (A), Dynamic Performance (D), Operating Temperature Capability (T), and Power Requirement (P). The original technical specifications and baseline linear Min–Max normalization were retained across all scenarios.

The group weighting coefficients were calculated from the normalized principal right eigenvectors of the corresponding pairwise comparison matrices. The resulting weights, consistency ratios, and three highest-ranked alternatives for each scenario are presented in Table 11.

The corresponding pairwise comparison matrices and detailed AHP consistency results are reported in Appendix A.

All three mission-oriented scenarios satisfied the adopted AHP consistency threshold of CR < 0.10. The consistency ratios were 0.0168 for long-duration INS, 0.0015 for UAV/tactical navigation, and 0.0189 for compact robotic systems. These values indicate acceptable internal consistency of the assumed pairwise judgments but do not establish the objective validity of the corresponding engineering preferences.

Under the long-duration INS scenario, Accuracy received the highest group weight (0.6143), reflecting the assumed importance of bias-related stability and stochastic noise for autonomous navigation. S-F120HA achieved the highest FOGPI score (0.8413), followed by S-F120HB (0.8405) and S-F120HC (0.8397). The increased emphasis on accuracy-related characteristics changed the highest-ranked alternative compared with the baseline configuration.

In the UAV/tactical navigation scenario, equal importance was assigned to Accuracy and Dynamic Performance, each receiving a group weight of 0.3936. DSP-1760 retained the highest position with a FOGPI score of 0.7936, followed by S-F120HA (0.7240) and S-F120HB (0.7235). This outcome reflects the combined contribution of accuracy-related and dynamic characteristics under the adopted weighting configuration.

Under the compact robotic systems scenario, Power Requirement received the greatest weight (0.4729), followed by Dynamic Performance (0.2844). VG910F1 became the highest-ranked device (0.8150), followed by VG103SD (0.7582) and VG1703 (0.7265). This ordering reflects the greater importance assigned to low power consumption and dynamic capability within the illustrative compact robotic preference structure.

These results demonstrate that different engineering preference structures can produce different highest-ranked alternatives even when the technical specifications, normalization procedure, and aggregation method remain unchanged. The AHP-derived weights represent illustrative engineering assumptions rather than empirically elicited expert judgments or experimentally validated mission requirements. Consequently, the resulting rankings should be interpreted as conditional comparative outcomes rather than confirmation of operational suitability for the corresponding applications.

#### 3.4.3. Global Weight-Space Sensitivity

The global sensitivity analysis examined simultaneous variations of all four functional-group weights using two Dirichlet sampling configurations: uniform sampling over the admissible weight simplex and baseline-centered sampling. Each configuration included 200,000 sampled weighting vectors.

The resulting first-place and top-three rank acceptability indices are summarized in Table 12.

Under uniform sampling of the admissible simplex, DSP-1760 occupied first place in 50.72% of the sampled weighting configurations, followed by VG910F1 (28.30%) and S-F120HA (11.71%). The remaining first-place frequencies were distributed among several other devices, demonstrating that the baseline leader does not retain its position throughout the investigated weighting space.

Under baseline-centered sampling, DSP-1760 occupied first place in 87.39% of the sampled configurations, whereas S-F120HA occupied first place in 12.61%. No other device occupied first place in the recorded baseline-centered configurations.

The top-three acceptability results further illustrate the influence of the sampling distribution. Under uniform sampling, DSP-1760 appeared among the three highest-ranked alternatives in 70.05% of configurations, compared with 52.23% for S-F120HA and 44.58% for VG910F1. Under baseline-centered sampling, S-F120HA and S-F120HB appeared among the top three in 99.99% and 99.78% of configurations, respectively.

The sampled weighting configurations therefore contain distinct preference regions associated with different highest-ranked devices. S-F120HA is favored when Accuracy receives greater importance, whereas VG910F1 benefits from configurations emphasizing Dynamic Performance and Power Requirement. DSP-1760 occupies a comparatively broad region associated with more balanced weighting configurations.

Together, the local, mission-oriented, and global analyses demonstrate that FOGPI rankings are conditional on the assumed weighting structure. The baseline ranking is relatively stable within the investigated baseline-centered preference region but does not remain invariant throughout the complete admissible weight space. The reported rank acceptability indices represent conditional frequencies under the specified sampling distributions rather than empirically established probabilities of engineering preferences.

### 3.5. Intercriterion Dependence and CRITIC-Based Weighting Results

The statistical dependencies among the six evaluation criteria and their influence on the FOGPI weighting structure were examined using Spearman rank-correlation analysis and the CRITIC method. The correlation analysis was used to identify potential redundancy among the selected characteristics, while CRITIC provided an alternative data-driven weighting configuration accounting for criterion variability and intercriterion relationships.

#### 3.5.1. Intercriterion Correlation Analysis

The pairwise Spearman rank-correlation coefficients calculated for the eleven investigated FOGs are presented in Table 13.

The strongest positive association was observed between Bias Stability (BS) and Angle Random Walk (ARW), with a Spearman coefficient of 0.922. This indicates that devices with relatively favorable BS values also tended to exhibit favorable ARW values within the investigated dataset.

Strong negative associations were identified between BS and Power Consumption (PC), with a coefficient of −0.806, and between Bandwidth (BW) and PC, with a coefficient of −0.779. These relationships reflect the characteristics of the investigated eleven-device sample rather than universal physical dependencies among FOG performance parameters.

The correlation between BS and ARW is particularly relevant to the baseline FOGPI weighting configuration, in which the two accuracy-related criteria jointly account for 50% of the total weight. Their statistical association indicates the possibility of partial redundancy in the weighted aggregation. However, BS and ARW describe physically distinct error characteristics, and their observed correlation does not establish that either criterion can be omitted without losing relevant information.

#### 3.5.2. CRITIC-Based Weighting Results

To examine the influence of statistical dependencies on the weighting structure, CRITIC-derived criterion weights were calculated using the same eleven-device decision matrix and baseline linear Min–Max normalization. The resulting weights were compared with the original engineering preference coefficients. The resulting CRITIC-derived weights and their comparison with the baseline weighting coefficients are presented in Table 14.

The combined weight assigned to BS and ARW decreased from 0.5000 in the baseline configuration to 0.3687 under CRITIC weighting. The ARW weight decreased from 0.2500 to 0.1370, whereas the BS weight decreased from 0.2500 to 0.2317. Conversely, the BW weight increased from 0.1250 to 0.1702, and the PC weight increased from 0.1000 to 0.1837.

The changes in weighting coefficients reflect the combined influence of normalized criterion variability and Pearson intercriterion correlations. The reduced combined weight of BS and ARW should not be attributed exclusively to their mutual association because CRITIC also accounts for relationships with the remaining criteria.

The resulting FOGPI scores and rankings obtained using CRITIC-derived weights are presented in Table 15.

Under CRITIC weighting, DSP-1760 retained the highest position, with its FOGPI score changing from 0.816965 to 0.771712. S-F120HA, S-F120HB, and S-F120HC also retained their second, third, and fourth positions, respectively.

More substantial changes occurred among the remaining devices. VG910F1 moved from seventh to fifth position, while GF120H-A decreased from sixth to ninth position. EG-1300 moved from fifth to sixth, whereas VG103SD and VG1703 improved to seventh and eighth positions, respectively. EG-200 and DSP-3000 retained the final two positions.

These results demonstrate that the weighting strategy affects both the relative contributions of the evaluation criteria and the ordering of individual alternatives. Although the highest-ranked device remained unchanged, several intermediate ranking positions were sensitive to the redistribution of criterion weights.

The CRITIC-derived coefficients represent data-dependent statistical weights rather than experimentally validated priorities for specific inertial-navigation applications. Consequently, the comparison should be interpreted as an assessment of weighting-method sensitivity and potential criterion redundancy rather than as independent validation of the baseline FOGPI ranking or evidence that CRITIC provides universally optimal weights.

### 3.6. Dataset-Composition and Input-Uncertainty Results

The sensitivity of FOGPI scores and rankings to changes in the comparison dataset and variations in the reported technical specifications was examined using leave-one-out (LOO) analysis and Monte Carlo simulation. These approaches investigate different sources of ranking variability: the LOO analysis evaluates the influence of dataset composition, whereas Monte Carlo simulation examines the effects of hypothetical perturbations in the input characteristics.

#### 3.6.1. Dataset-Composition Sensitivity

The LOO analysis was performed by removing each of the eleven FOGs individually and recalculating the normalized criteria, FOGPI scores, and rankings for the remaining ten devices. The resulting rank correlations and highest-ranked alternatives are presented in Table 16.

For ten of the eleven LOO scenarios, the relative ordering of the remaining FOGs was unchanged, corresponding to Spearman’s ρ and Kendall’s τ values of 1.000. When DSP-1760 was removed, S-F120HA became the highest-ranked remaining device, while the relative ordering of the other alternatives remained unchanged.

A different result was observed when DSP-3000 was excluded. Spearman’s ρ decreased to 0.915 and Kendall’s τ to 0.822, and S-F120HA replaced DSP-1760 as the highest-ranked device. The first four positions changed from DSP-1760–S-F120HA–S-F120HB–S-F120HC to S-F120HA–S-F120HB–S-F120HC–DSP-1760.

This change was associated with the influence of DSP-3000 on the ARW normalization bound. In the original dataset, DSP-3000 defines the maximum ARW value of 0.067°/√h. Its exclusion reduces the maximum observed ARW to 0.040°/√h, modifying the normalized ARW values of the remaining devices. Consequently, the relative contribution of accuracy-related characteristics changes despite the technical specifications of the remaining FOGs being unchanged.

These findings demonstrate that the baseline FOGPI ranking is not invariant to changes in the comparison dataset. In particular, alternatives defining extreme criterion values may influence the relative ordering of other devices through sample-dependent normalization.

#### 3.6.2. Input-Uncertainty Analysis

The Monte Carlo analysis examined the influence of hypothetical variations in manufacturer-reported specifications on the resulting FOGPI scores and rankings. Three relative perturbation levels (±5%, ±10%, and ±20%) were investigated using 100,000 iterations per perturbation level and normalization configuration.

The ±10% perturbation scenario was selected for detailed presentation. The corresponding results for baseline linear Min–Max normalization are summarized in Table 17.

Under the ±10% scenario, DSP-1760 occupied first place in 39.28% of the simulated iterations, compared with 19.85%, 19.59%, and 19.24% for S-F120HA, S-F120HB, and S-F120HC, respectively. Although DSP-1760 retained the highest mean FOGPI score (0.7548), the first-place frequencies indicate that the deterministic baseline ordering was sensitive to the assumed input variations.

The simulated score distributions of the three S-F120H models exhibited substantial overlap. Their mean FOGPI scores ranged from 0.7300 to 0.7311, while the corresponding 2.5th–97.5th percentile intervals overlapped extensively. Thus, their small deterministic score differences did not lead to clearly separated score distributions under the investigated perturbation model.

**Figure 7:**
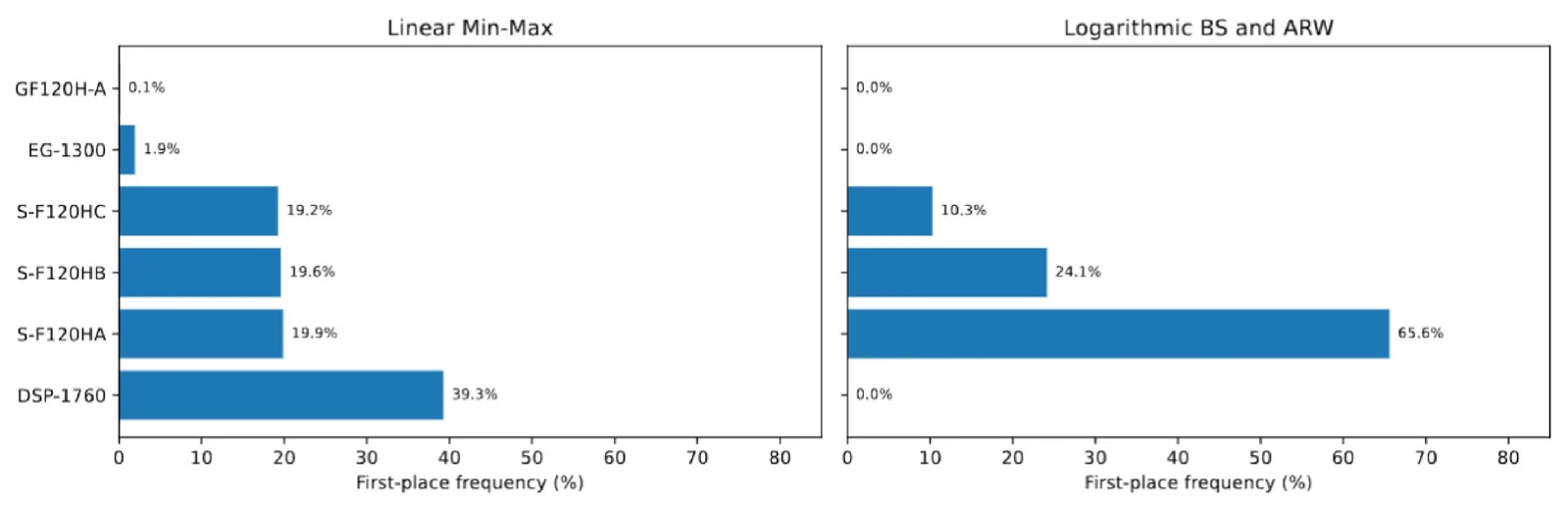
First-place rank frequencies under independent uniform ±10% perturbations of the input specifications for linear and logarithmic FOGPI normalization (100,000 iterations per configuration). Only devices with a nonzero recorded first-place frequency in at least one normalization configuration are displayed.

The influence of normalization was also examined by comparing linear Min–Max normalization with logarithmic normalization of BS and ARW. Under logarithmic normalization and ±10% perturbations, S-F120HA occupied first place in 65.61% of the iterations, followed by S-F120HB (24.13%) and S-F120HC (10.27%). DSP-1760 did not occupy first place in the recorded iterations under this configuration. The corresponding first-place rank frequencies under both normalization approaches are illustrated in Figure 7.

Across the three perturbation levels, the first-place frequency of DSP-1760 under linear normalization decreased from 46.38% at ±5% to 39.28% at ±10% and 34.32% at ±20%. Under logarithmic normalization, the corresponding first-place frequencies of S-F120HA were 75.05%, 65.61%, and 61.37%.

These results demonstrate that the ranking is sensitive to both the assumed magnitude of input variations and the selected normalization procedure. However, the simulated frequencies and score intervals are conditional on the hypothetical independent uniform perturbation model. They do not represent experimentally established measurement uncertainties, manufacturer production variability, or formal metrological confidence intervals.

Taken together, the LOO and Monte Carlo analyses demonstrate two distinct limitations of the baseline comparative ranking. The LOO results reveal dependence on the composition of the comparison dataset, whereas the Monte Carlo results show that the ordering may vary under assumed perturbations of the technical specifications. Consequently, ranking stability should be evaluated with respect to the specific source of variability being investigated rather than interpreted as a universal property of FOGPI.

### 3.7. Cross-Method Comparison with TOPSIS and VIKOR

To examine the dependence of the comparative ranking on the selected multi-criteria decision-making method, the eleven investigated FOGs were evaluated using FOGPI, TOPSIS, and VIKOR. All three approaches were applied to the same decision matrix, evaluation criteria, and baseline weighting coefficients. The resulting scores and rankings are presented in Table 18.

The comparison revealed both agreement and differences among the three methods. FOGPI and VIKOR produced identical complete rankings, with DSP-1760 occupying the first position, followed by S-F120HA, S-F120HB, S-F120HC, and EG-1300. In contrast, TOPSIS produced a different ordering, particularly among the highest-ranked devices.

Under TOPSIS, EG-1300 achieved the highest relative closeness coefficient (0.7982), whereas DSP-1760 ranked fifth (0.7787). This contrasts with their positions under FOGPI and VIKOR, where DSP-1760 ranked first and EG-1300 fifth. The three S-F120H models retained positions two to four under all three methods.

To further examine the difference between EG-1300 and DSP-1760, their distances from the positive and negative ideal solutions were calculated using the weighted vector-normalized decision matrix. EG-1300 exhibited a positive ideal distance of 0.057378 and a negative ideal distance of 0.226910, resulting in a relative closeness coefficient of 0.798169. The corresponding distances for DSP-1760 were 0.057637 and 0.202769, yielding a relative closeness coefficient of 0.778665.

Although DSP-1760 exhibited more favorable Maximum Input Rate, Operating Temperature Range Width, and Power Consumption characteristics, EG-1300 benefited from its lower Bias Stability and Angle Random Walk values. These accuracy-related advantages contributed to its greater separation from the negative ideal solution. Consequently, EG-1300 achieved a higher TOPSIS relative closeness coefficient despite occupying a lower position under the weighted additive FOGPI approach. This result illustrates how differences in the distribution of criterion-level performance influence the final ranking under the Euclidean distance-based TOPSIS method.

To quantify the agreement among the rankings, Spearman’s rank correlation coefficient and Kendall’s rank correlation coefficient were calculated. The resulting values are summarized in Table 19.

FOGPI and VIKOR exhibited identical complete rankings, with Spearman’s ρ and Kendall’s τ both equal to 1.000. FOGPI and TOPSIS showed lower rank agreement, with ρ = 0.818 and τ = 0.636. The same coefficients were obtained for TOPSIS and VIKOR because FOGPI and VIKOR produced identical rankings.

The agreement between FOGPI and VIKOR can be partly explained by a structural relationship between their aggregation procedures. Under the adopted linear Min–Max normalization and identical criterion weights, the VIKOR group-utility measure satisfies

*S_i_* = 1 − *FOGPI_i_* (49)

Therefore, the FOGPI ranking and the ranking based on the VIKOR group-utility component are necessarily identical. However, the final VIKOR compromise index also incorporates the maximum individual-regret measure *R_i_*. With the compromise parameter set to *v* = 0.5, complete agreement between the final VIKOR ranking and FOGPI is not mathematically guaranteed. The observed agreement indicates that the individual-regret contribution did not cause rank reversals relative to the group-utility ordering for the investigated dataset.

The cross-method comparison therefore demonstrates methodological dependence rather than independent validation of FOGPI. Because all three methods use the same alternatives, criteria, and weighting coefficients, agreement among their rankings cannot establish the physical correctness or superiority of the proposed index. The different ordering produced by TOPSIS further illustrates that the relative evaluation of FOGs is conditional on the selected aggregation principle.

### 3.8. Application-Specific Admissibility Results

The application-specific admissibility assessment was performed to examine the limitations of unrestricted FOGPI ranking when mandatory technical requirements must be satisfied. Two complementary evaluations were conducted: temperature-based screening using the absolute operating-temperature limits of the investigated devices and a two-stage selection procedure combining application-specific technical constraints with subsequent FOGPI ranking.

#### 3.8.1. Temperature-Based Admissibility

The temperature-based assessment was conducted using the manufacturer-reported minimum and maximum operating temperatures of the eleven investigated FOGs. Two illustrative operating scenarios were considered: extended cold operation (−45 to +65 °C) and extended upper-temperature operation (−40 to +75 °C). The corresponding operating-temperature intervals, range widths, and admissibility results are summarized in Table 20.

Under the extended cold-operation scenario, only S-F120HA, S-F120HB, and S-F120HC satisfied the required temperature interval. Their reported operating range of −45 to +70 °C fully covered the required interval of −45 to +65 °C. The remaining eight devices did not satisfy the lower-temperature requirement.

In contrast, under the extended upper-temperature scenario, DSP-1760, EG-200, and DSP-3000 satisfied the required interval of −40 to +75 °C. The S-F120H models did not satisfy this scenario because their maximum operating temperature was +70 °C, despite having the same TRW value of 115 °C as DSP-1760.

These results demonstrate that identical operating-temperature range widths do not necessarily imply equivalent suitability for a specified thermal environment. In particular, the S-F120H series and DSP-1760 have the same TRW but different absolute temperature limits, resulting in different admissibility outcomes.

The examined technical documentation also contained selected temperature-dependent accuracy characteristics. For example, the DSP-3000 digital configuration specifies a bias variation over temperature of ≤3°/h (1σ) and scale-factor variation of ≤250 ppm (1σ). EG-1300 reports a typical bias-over-temperature value of 0.1°/h and compensated scale-factor variation of ≤50 ppm (1σ). However, the definitions and reporting conditions of these characteristics were not sufficiently homogeneous to incorporate them as common quantitative criteria for all eleven devices.

The results indicate that separate minimum and maximum operating-temperature limits provide more application-specific information than TRW alone. Nevertheless, satisfaction of the specified operating-temperature interval does not establish that gyroscope accuracy remains constant throughout that interval.

#### 3.8.2. Application-Specific Two-Stage Selection

The two-stage selection procedure was evaluated using two illustrative engineering scenarios: a high-accuracy inertial navigation system (INS) and a compact unmanned aerial vehicle (UAV). In each scenario, the eleven FOGs were first assessed against mandatory accuracy, dynamic-performance, power-consumption, and operating-temperature requirements. Only devices satisfying all specified constraints were retained for subsequent FOGPI ranking.

The resulting admissible alternatives and their highest-ranked devices are summarized in Table 21.

For the high-accuracy INS scenario, five gyroscopes satisfied all mandatory requirements: S-F120HA, S-F120HB, S-F120HC, GF120H-A, and EG-1300. Among these alternatives, S-F120HA achieved the highest baseline FOGPI score (0.791469). Although DSP-1760 occupied first place in the unrestricted baseline ranking, it was excluded because its reported BS (0.050°/h) and ARW (0.012°/√h) exceeded the specified accuracy limits.

The admissibility of GF120H-A in the illustrative INS scenario is conditional on the provisional bandwidth and power-consumption values used in the decision matrix. Both values coincide with the corresponding admissibility thresholds; therefore, the model’s eligibility cannot be considered independently verified from the available technical documentation.

For the compact UAV scenario, three devices satisfied the mandatory requirements: EG-200, VG103SD, and VG910F1. Among these alternatives, VG910F1 achieved the highest FOGPI score (0.567114), followed by VG103SD (0.530271) and EG-200 (0.421713). DSP-1760 was excluded because its reported power consumption of 3 W exceeded the specified 2 W limit, despite achieving the highest score in the unrestricted baseline ranking.

The two-stage procedure therefore produced different preferred alternatives for the investigated engineering scenarios. By applying mandatory constraints before ranking, it prevented favorable values in other criteria from compensating for failure to satisfy application-specific requirements.

The scenarios represent illustrative engineering requirements rather than standardized qualification profiles or experimentally validated mission specifications. In particular, the compact UAV scenario did not incorporate additional installation constraints such as mass, dimensions, interfaces, or platform-specific environmental qualification. Consequently, the resulting selections should be interpreted as conditional decision-support outcomes rather than confirmation of operational suitability for a particular system.

## 4. Discussion

The results presented in the previous section demonstrate the applicability of the proposed Fiber Optic Gyroscope Performance Index (FOGPI) for the comparative evaluation of FOGs with different performance characteristics. This section discusses the obtained results from the perspective of the proposed evaluation methodology, compares the findings with established multi-criteria approaches and existing studies reported in the literature, and considers the practical implications, limitations, and potential directions for further development of the proposed framework.

### 4.1. Methodological Interpretation of FOGPI

The proposed Fiber Optic Gyroscope Performance Index (FOGPI) provides a structured approach for comparing selected technical characteristics of fiber-optic gyroscopes within a common multi-criteria evaluation framework. By integrating six specification-based attributes into four functional groups, the methodology enables heterogeneous accuracy-related, dynamic, operating-temperature, and power-related characteristics to be evaluated under an explicitly defined preference structure. Its methodological value lies in the transparency of the evaluation procedure and in the systematic investigation of how different modeling assumptions influence the resulting scores and rankings [68,69].

The present study does not introduce a fundamentally new mathematical aggregation operator. Instead, it applies established normalization and weighted aggregation principles to a FOG-specific decision problem and investigates the conditions under which comparative rankings remain stable or change. In particular, the results demonstrate that normalization functions, weighting configurations, intercriterion dependencies, input-specification variations, and comparison-set composition can influence the relative ordering of the investigated devices. These dependencies are fundamental properties of specification-based multi-criteria evaluation rather than secondary numerical effects.

FOGPI should therefore be interpreted as a conditional comparative index rather than an intrinsic measure of gyroscope quality. A high score indicates a favorable combination of the six selected characteristics under the adopted normalization procedure, weighting configuration, and comparison dataset. It does not establish universal superiority or confirm suitability for every navigation application. This distinction is particularly important when technical characteristics are obtained from heterogeneous manufacturer documentation rather than standardized experimental measurements.

The compensatory nature of weighted additive aggregation further limits the interpretation of unrestricted FOGPI rankings. Favorable characteristics may offset weaker values in other criteria, even when the latter represent mandatory technical requirements. Accordingly, the proposed framework is most appropriately applied as a decision-support procedure in which aggregate ranking is complemented by explicit admissibility constraints and application-specific engineering judgments. FOGPI therefore supports the analysis of technical trade-offs rather than replacing detailed sensor qualification or system-level performance assessment.

### 4.2. Interpretation of Ranking Sensitivity and Engineering Trade-Offs

The baseline FOGPI ranking illustrates how the combined contribution of accuracy-related, dynamic, operating-temperature, and power-related characteristics influences the comparative evaluation of fiber-optic gyroscopes. Under the reference weighting configuration and linear Min–Max normalization, DSP-1760 achieved the highest aggregate score (0.816965), although the S-F120H series exhibited more favorable reported Bias Stability (BS) and Angle Random Walk (ARW) characteristics. This result reflects the compensatory nature of weighted additive aggregation, in which favorable performance across several criteria can offset less favorable values in others. Consequently, the highest aggregate score should not be interpreted as evidence of superior accuracy or universal suitability for inertial-navigation applications.

The normalization sensitivity analysis (Section 3.3) demonstrates that the ranking is strongly influenced by the mathematical representation of accuracy-related characteristics. Replacing linear Min–Max normalization with logarithmic normalization for BS and ARW changed the highest-ranked alternative from DSP-1760 to S-F120HA, while DSP-1760 moved from first to fourth position. This change reflects the greater emphasis placed on multiplicative differences between reported accuracy-related values under logarithmic scaling. The fixed-reference analysis further showed that eliminating sample-dependent normalization does not produce a unique or universally preferable ranking: different reference intervals and transformation functions can favor different performance profiles. Thus, normalization should be regarded as a substantive modeling assumption rather than a neutral preprocessing operation.

The influence of weighting preferences was also evident in the local and global sensitivity analyses (Section 3.4). In the local one-factor-at-a-time analysis, increasing the Accuracy group weight beyond approximately 0.592, while proportionally rescaling the remaining weights, produced a change in the highest-ranked alternative from DSP-1760 to S-F120HA. The global weight-space analysis showed that DSP-1760 occupied first place in 50.72% of uniformly sampled weighting configurations and 87.39% of baseline-centered configurations. These frequencies indicate that ranking stability depends on the investigated region of the preference space and should not be interpreted as empirically established probabilities of engineering preferences.

The mission-oriented AHP scenarios (Section 3.4.2) provide a complementary illustration of this preference dependence. Under the revised long-duration INS weighting configuration, Accuracy received a group weight of 0.6143, and S-F120HA became the highest-ranked alternative. DSP-1760 retained first place in the UAV/tactical navigation scenario, where Accuracy and Dynamic Performance were assigned equal weights of 0.3936. In contrast, VG910F1 achieved the highest score in the compact robotic systems scenario, in which Power Requirement received the greatest weight (0.4729). These outcomes illustrate how changes in assumed engineering priorities can alter the preferred alternative without changing the underlying technical specifications. However, the AHP weights were derived from illustrative pairwise judgments rather than experimentally validated mission requirements.

The intercriterion correlation analysis (Section 3.5) identified a strong positive Spearman association between BS and ARW (ρ = 0.922), suggesting potential partial redundancy within the Accuracy group. Although these characteristics describe physically different error processes, their statistical association can influence the interpretation of their combined contribution to an additive index. The CRITIC-based analysis provided an alternative weighting structure that reduced the combined weight of BS and ARW from 0.5000 to 0.3687. While DSP-1760 remained the highest-ranked device, several intermediate positions changed, demonstrating that criterion variability and intercriterion correlations can influence comparative rankings. Nevertheless, statistical weighting does not establish the physical importance of individual characteristics for specific navigation applications.

The dataset-composition and input-uncertainty analyses (Section 3.6) revealed additional sources of ranking variability. In the leave-one-out analysis, excluding DSP-3000 changed the observed maximum ARW value and altered the ranking of the remaining devices, with S-F120HA replacing DSP-1760 as the highest-ranked alternative. The Monte Carlo analysis further demonstrated that hypothetical perturbations of manufacturer-reported specifications can produce substantial changes in ranking frequencies, particularly among devices with closely spaced deterministic scores. These findings indicate that the apparent precision of an aggregate FOGPI value should not be confused with certainty about the corresponding ranking position.

The application-specific admissibility results (Section 3.8) demonstrate why unrestricted FOGPI rankings must be distinguished from requirement-based sensor selection. Devices with identical Operating Temperature Range Width values may have different absolute operating-temperature limits and therefore satisfy different environmental requirements. Furthermore, under the illustrative high-accuracy INS scenario, DSP-1760 was excluded because its reported BS and ARW values exceeded the mandatory accuracy thresholds, while S-F120HA achieved the highest score among admissible devices. Under the compact UAV scenario, VG910F1 became the highest-ranked admissible alternative because the baseline leader did not satisfy the specified power-consumption constraint. These results show that favorable aggregate scores cannot compensate for failure to satisfy mandatory engineering requirements.

Taken together, the findings indicate that FOGPI is most informative when interpreted alongside normalization sensitivity, weighting assumptions, input-data limitations, and application-specific constraints. The framework supports transparent comparison of selected gyroscope characteristics, but its rankings should be understood as conditional engineering assessments rather than universal measures of sensor superiority.

### 4.3. Comparison with Existing Research and MCDM Approaches

Previous research on fiber-optic gyroscopes has extensively investigated improvements in sensor architectures, bias-related stability, angle random walk, scale-factor characteristics, and temperature-dependent performance through optical design optimization, error compensation, and signal-processing techniques [70,71,72,73,74]. These studies provide important insights into individual performance characteristics and the physical mechanisms governing gyroscope operation. However, the present investigation addresses a different methodological problem: how heterogeneous manufacturer-reported specifications can be integrated into a transparent comparative evaluation framework and how the resulting rankings depend on the assumptions adopted during multi-criteria aggregation.

Established multi-criteria decision-making (MCDM) methods, including the Analytic Hierarchy Process (AHP), TOPSIS, and VIKOR, provide general procedures for assigning criterion weights, comparing alternatives, and constructing preference rankings [75,76,77]. The proposed FOGPI framework does not replace these approaches or introduce a fundamentally new mathematical aggregation principle. Instead, it applies weighted additive aggregation to six selected FOG characteristics and systematically examines the influence of normalization, weighting preferences, intercriterion dependencies, comparison-set composition, and input-specification uncertainty on the resulting scores and rankings.

The cross-method comparison presented in Section 3.7 demonstrated that FOGPI and VIKOR produced identical complete rankings for the eleven investigated devices, whereas TOPSIS yielded a different ordering. This agreement should be interpreted in light of the mathematical relationship between FOGPI and the VIKOR group-utility measure. Under identical criterion weights and the adopted linear Min–Max normalization, the VIKOR group-utility measure satisfies *S_i_* = 1 − *FOGPI_i_*. Therefore, agreement between FOGPI and the group-utility component is mathematically determined rather than independent evidence supporting the validity of the proposed index. The final VIKOR compromise ranking also incorporates individual regret, and its agreement with FOGPI is conditional on the investigated dataset and weighting configuration.

The differences observed under TOPSIS further illustrate the influence of the aggregation principle. Whereas FOGPI combines normalized criterion values additively, TOPSIS evaluates alternatives through their weighted Euclidean distances from positive and negative ideal solutions. Consequently, devices exhibiting different combinations of favorable and unfavorable characteristics may receive different relative positions. In the investigated dataset, EG-1300 ranked first under TOPSIS but fifth under FOGPI, while DSP-1760 ranked first under FOGPI but fifth under TOPSIS. These differences demonstrate that the resulting ranking is method-dependent even when the same technical specifications, criteria, and weighting coefficients are used.

The present findings also reinforce the importance of interpreting accuracy-related characteristics within their measurement and reporting contexts. Previous studies emphasize the relevance of bias-related stability and stochastic noise for inertial-navigation performance [78,79]. However, the specification audit conducted in this study identified differences in the statistical definitions, measurement conditions, and operating configurations associated with manufacturer-reported characteristics. Thus, integrating BS and ARW into a common comparative index does not establish their strict metrological equivalence across devices. This distinction limits the extent to which aggregate rankings can be interpreted as physically validated differences in navigation performance.

Taken together, the comparison with existing research and established MCDM methods indicates that the principal contribution of the proposed framework lies in its FOG-specific evaluation structure and systematic examination of methodological sensitivity rather than in the introduction of a new aggregation algorithm. The framework provides a reproducible basis for identifying ranking changes associated with alternative modeling assumptions while emphasizing the need for specification traceability, application-specific requirements, and cautious interpretation of comparative scores.

### 4.4. Limitations and Future Research Directions

Several limitations should be considered when interpreting the results of the proposed FOGPI framework. These limitations arise primarily from the heterogeneity of manufacturer-reported specifications, the restricted set of evaluation criteria, the assumptions underlying normalization and weighting, and the absence of independent experimental validation. Although the sensitivity analyses provide quantitative information about ranking variability, they do not eliminate these limitations or establish the physical superiority of any investigated device.

First, the evaluation was based on publicly available technical specifications rather than measurements obtained under a common experimental protocol. As demonstrated by the specification audit in Section 3.1.2, the investigated manufacturers reported bias-related stability, angle random walk, bandwidth, and power consumption using different statistical definitions, measurement conditions, and operating configurations. In particular, bias-related characteristics may represent different statistical quantities, while power-consumption specifications include typical, maximum, steady-state, and incompletely defined values. Consequently, numerical differences between reported characteristics do not necessarily correspond to directly comparable differences in experimentally measured sensor performance. The resulting FOGPI scores should therefore be interpreted as conditional comparisons of published specifications rather than independently verified measures of gyroscope quality.

An additional source-related limitation concerns GF120H-A. Although the available model-specific documentation provides bias-related, angle random walk, maximum input rate, and operating-temperature specifications, its bandwidth and power-consumption values could not be independently verified for the exact model configuration. These values were retained as provisional inputs in the decision matrix. Furthermore, the source documentation contains two reported zero-bias stability limits whose interpretation requires consideration of their stated measurement conditions. The uncertainty associated with these specifications may affect the device’s comparative score and its admissibility under application-specific requirements. The hypothetical perturbation model employed in the Monte Carlo analysis does not resolve such source-verification limitations.

Second, the study examined a heterogeneous but limited sample of eleven commercial FOG models. The dataset was selected to investigate the methodological behavior of the proposed framework rather than to represent the complete commercial FOG market. Consequently, the observed rankings, intercriterion correlations, and CRITIC-derived weights may change when additional devices or different performance classes are included. The leave-one-out analysis in Section 3.6.1 demonstrated this dependence: excluding DSP-3000 changed the maximum observed ARW value and altered the ordering of the remaining devices. Therefore, the numerical results should not be generalized to all commercially available FOGs.

Third, the FOGPI formulation includes only six technical characteristics grouped into four functional categories. Although these attributes represent relevant accuracy-related, dynamic, operating-temperature, and energy-related properties, they do not provide a complete description of gyroscope performance or system-level integration requirements. Other characteristics, including scale-factor stability and nonlinearity, bias repeatability, acceleration sensitivity, vibration and shock resistance, magnetic sensitivity, long-term reliability, startup and warm-up time, mass, dimensions, and communication interfaces, may be essential for specific applications. These parameters were excluded because sufficiently complete and consistently defined specifications were unavailable across the investigated dataset. Their omission limits the applicability of the index to engineering decisions requiring a broader set of technical requirements.

Fourth, the normalization procedure introduces assumptions that influence the resulting scores and rankings. The baseline linear Min–Max normalization is dependent on the minimum and maximum values observed within the comparison dataset, whereas logarithmic normalization emphasizes multiplicative differences in accuracy-related characteristics. The analyses in Section 3.3 demonstrated that changing the normalization function can alter the highest-ranked device without changing the technical specifications or weighting coefficients. Fixed-reference normalization eliminates changes in the numerical scores of unchanged devices caused solely by changes in comparison-set composition, provided that the reference bounds remain constant. However, its results remain dependent on the selected reference intervals and transformation functions. Because the adopted reference bounds were illustrative rather than experimentally calibrated, fixed-reference FOGPI scores should not be interpreted as universally standardized performance measures.

Fifth, the weighting coefficients represent assumed engineering preferences rather than objectively established physical priorities. The baseline weighting configuration was not derived from experimentally measured navigation-error budgets, formal mission requirements, or structured expert elicitation. Although the local and global sensitivity analyses in Section 3.4 quantified the influence of weighting variations, they cannot determine a universally optimal preference structure. Similarly, the mission-oriented AHP scenarios were constructed from illustrative pairwise judgments, and acceptable consistency ratios establish only their internal consistency. The CRITIC-derived weights examined in Section 3.5 account for statistical variability and intercriterion correlations but remain dependent on the investigated decision matrix. Consequently, neither AHP nor CRITIC provides independent physical validation of the adopted weighting coefficients.

Sixth, the input-uncertainty analysis was based on hypothetical independent uniform perturbations of manufacturer-reported specifications. The investigated ±5%, ±10%, and ±20% scenarios were selected to examine the sensitivity of ranking outcomes rather than to represent empirically established uncertainty distributions. The resulting Monte Carlo score intervals and rank frequencies are therefore conditional on the assumed perturbation model and should not be interpreted as formal metrological confidence intervals or experimentally verified probabilities of device superiority. More realistic uncertainty assessment would require manufacturer-specific statistical information, repeated measurements, and consideration of dependencies among error sources and operating conditions.

A further limitation concerns the compensatory nature of weighted additive aggregation. Although FOGPI allows trade-offs among normalized characteristics, a favorable aggregate score does not guarantee satisfaction of mandatory application requirements. The two-stage admissibility procedure in Section 3.8 addresses this issue by excluding alternatives that fail specified technical constraints before ranking the remaining devices. Nevertheless, the thresholds adopted for the illustrative INS and compact UAV scenarios were not derived from formally documented mission specifications. Furthermore, the temperature-based assessment used manufacturer-reported operating limits and did not establish that bias stability, angle random walk, or scale-factor characteristics remain unchanged across the specified temperature intervals. The admissibility results therefore demonstrate a decision procedure rather than certify device suitability for particular operational platforms.

Finally, the comparison with TOPSIS and VIKOR should not be regarded as independent validation of FOGPI. All three methods were applied to the same decision matrix and weighting configuration, and the agreement between FOGPI and the VIKOR group-utility component follows from their mathematical relationship under the adopted normalization. The observed agreement or disagreement among complete rankings therefore characterizes methodological dependence rather than the physical correctness of any particular ranking method.

Future research should prioritize experimental evaluation of representative FOG devices under standardized measurement conditions, including consistent temperature settings, averaging intervals, signal-processing configurations, and dynamic test procedures. Such investigations would support more rigorous assessment of specification comparability and provide independent evidence for evaluating the practical relevance of the proposed rankings. Further development should also consider expanding the decision matrix to include additional characteristics when sufficiently comparable data become available, incorporating manufacturer-specific uncertainty models, and evaluating larger and more diverse sets of devices.

Another important direction is the development of application-specific weighting coefficients, reference bounds, and utility functions derived from documented system requirements, navigation-error budgets, or structured expert judgments. Such extensions could improve the physical interpretability and transferability of the proposed framework. Future investigations should also examine the integration of environmental performance characteristics and mandatory system-level constraints into practical sensor-selection workflows, while preserving the distinction between comparative scoring and formal device qualification.

## 5. Conclusions

This study developed and systematically evaluated the Fiber Optic Gyroscope Performance Index (FOGPI), a six-criterion, specification-based framework for the comparative assessment of commercially available fiber-optic gyroscopes. The proposed index integrates Bias Stability, Angle Random Walk, Maximum Input Rate, Bandwidth, Operating Temperature Range Width, and Power Consumption into a single dimensionless score using normalized technical characteristics and explicitly defined weighting coefficients. The principal contribution of the study lies not in introducing a new mathematical aggregation operator, but in systematically examining the methodological assumptions that influence the interpretation, stability, and applicability of multi-criteria FOG rankings.

The framework was applied to eleven commercial FOG models with heterogeneous reported performance characteristics. Under the baseline linear Min–Max normalization and reference weighting configuration, DSP-1760 achieved the highest FOGPI score (0.816965), followed by the S-F120H series. However, the analyses demonstrated that the resulting ranking is conditional on the adopted normalization and weighting assumptions. Logarithmic normalization of Bias Stability and Angle Random Walk changed the highest-ranked device to S-F120HA, while increasing the Accuracy group weight above approximately 0.592 produced a similar rank reversal. The mission-oriented AHP scenarios further demonstrated that different assumed engineering priorities can favor different devices, including S-F120HA for long-duration inertial navigation, DSP-1760 for UAV/tactical navigation, and VG910F1 for compact robotic systems.

The robustness analyses identified additional sources of ranking variability. Global weight-space sampling demonstrated that the baseline leader does not retain first place throughout the complete admissible preference space, while leave-one-out analysis revealed that sample-dependent Min–Max normalization can alter the relative ordering of unchanged devices. Fixed-reference normalization eliminated changes in individual numerical scores caused solely by modifications to the comparison dataset, although the resulting scores remained dependent on the selected reference bounds and transformation functions. The Monte Carlo analysis further showed that hypothetical variations in manufacturer-reported specifications can substantially influence rank frequencies, particularly among devices with closely spaced deterministic scores.

The intercriterion analysis identified statistical dependencies among the selected characteristics, including a strong positive association between Bias Stability and Angle Random Walk. CRITIC-based weighting demonstrated that accounting for criterion variability and intercriterion correlations can modify the weighting structure and intermediate ranking positions. Benchmarking against TOPSIS and VIKOR revealed differences associated with the selected aggregation principle. Although FOGPI and VIKOR produced identical complete rankings for the investigated dataset, this agreement does not constitute independent validation because of the mathematical relationship between the FOGPI aggregation procedure and the VIKOR group-utility measure.

The application-specific admissibility assessment demonstrated that unrestricted aggregate rankings should not replace mandatory technical screening. Devices with identical Operating Temperature Range Width values may have different absolute temperature limits, and the highest-ranked device under the baseline FOGPI configuration may fail application-specific accuracy or power-consumption requirements. Accordingly, combining comparative ranking with explicit admissibility constraints provides a more appropriate decision-support procedure for engineering sensor selection.

The principal limitations of the study arise from the use of heterogeneous manufacturer-reported specifications, the restricted eleven-device dataset, and the absence of independent experimental validation. The calculated FOGPI scores therefore represent conditional comparisons of selected published characteristics rather than absolute measures of gyroscope quality or verified operational suitability. Future research should prioritize standardized experimental characterization, improved specification traceability, broader evaluation datasets, and application-specific weighting and reference scales derived from documented system requirements and navigation-error budgets.

Overall, the proposed FOGPI framework provides a transparent and reproducible approach for examining engineering trade-offs among selected FOG characteristics. Its practical value lies in supporting structured comparative evaluation while making the influence of normalization, weighting, input-data limitations, and mandatory application requirements explicit.

## Figures and Tables

**Table 1:** Technical characteristics and data sources of the investigated FOGs.

Gyroscope	Manufacturer/Source	Class	BS (°/h)	ARW (°/√h)	MIR (°/s)	BW (Hz)	TRW (°C)	PC (W)
S-F120HA	Shenzhen FOVA Technology Co., Ltd., Shenzhen, China [47]	Navigation-oriented	0.001	0.0002	400	200	115	4
S-F120HB	Shenzhen FOVA Technology Co., Ltd., Shenzhen, China [47]	Navigation-oriented	0.002	0.0003	400	200	115	4
S-F120HC	Shenzhen FOVA Technology Co., Ltd., Shenzhen, China [47]	Navigation-oriented	0.003	0.0004	400	200	115	4
GF120H-A	Micro-Magic Inc., Hangzhou, China [38]	Navigation-oriented	0.004	0.0020	500	100	105	6
EG-1300	EMCORE Corporation, Tinley Park, IL, USA [39]	Navigation-oriented	0.010	0.0020	300	500	110	5
DSP-1760	KVH Industries, Bristol, RI, USA [41]	Tactical-oriented	0.050	0.0120	490	440	115	3
EG-200	EMCORE Corporation, Tinley Park, IL, USA [42]	Tactical-oriented	1.000	0.0400	300	300	115	1.3
DSP-3000	EMCORE Corporation, Tinley Park, IL, USA [43]	Tactical-oriented	1.000	0.0670	375	440	115	1.25
VG103SD	MostaTech Ltd., Mosta, Malta [45]	Compact	1.000	0.0150	400	600	110	0.5
VG1703	MostaTech Ltd., Mosta, Malta [44]	Compact	1.000	0.0080	130	1000	110	0.5
VG910F1	MostaTech Ltd., Mosta, Malta [46]	Compact	1.000	0.0200	400	1000	110	0.5

Note: BS—Bias Stability; ARW—Angle Random Walk; MIR—Maximum Input Rate; BW—Bandwidth; TRW—Operating Temperature Range Width; PC—Power Consumption. TRW was calculated as *TRW* = *T_max_* − *T_min_* using the reported operating-temperature limits. The reported BS and ARW specifications may differ in statistical definitions and measurement conditions, as detailed in the specification audit. Power-consumption values refer to individual FOG units or specified single-axis configurations rather than complete multi-axis inertial measurement units. Because the reported PC values represent different operating conditions (typical, maximum, steady-state, or unspecified), they are not necessarily directly comparable. For DSP-1760, the reported 3 W corresponds to typical power consumption of the single-axis housed configuration (6 W maximum). For GF120H-A, the BW and PC values remain provisional because model-specific confirmation was unavailable. The available documentation also reports two zero-bias stability limits, ≤0.010°/h (1σ, 10 s) and ≤0.004°/h (1σ, 100 s). The latter value was used in the decision matrix as a manufacturer-reported specification limit.

**Table 2:** Weight coefficients of criteria groups.

Group of Criteria	Weight
Accuracy	0.50
Dynamic Performance	0.25
Operating Temperature Capability	0.15
Power Requirement	0.10

**Table 3:** Weight coefficients of individual criteria.

Criterion	Designation	Weight
Bias Stability	BS	0.25
Angle Random Walk	ARW	0.25
Maximum Input Rate	MIR	0.125
Bandwidth	BW	0.125
Operating Temperature Range Width	TRW	0.15
Power Consumption	PC	0.10

**Table 4:** Fixed reference bounds for the six FOG evaluation criteria.

Criterion	Lower Bound *L_j_*	Upper Bound *U_j_*	Direction
BS (°/h)	0.0001	10	Cost
ARW (°/√h)	0.0001	0.1	Cost
MIR (°/s)	0	1000	Benefit
BW (Hz)	0	1000	Benefit
TRW (°C)	0	150	Benefit
PC (W)	0	10	Cost

Note: The reference intervals are illustrative engineering anchors adopted for methodological comparison. They are not universal physical limits, IEEE-defined performance thresholds, or experimentally validated application requirements.

**Table 5:** Traceability, statistical definitions, and reporting conditions of the investigated FOG specifications.

Gyroscope	Source	BS Definition and Conditions	ARW Definition and Conditions	MIR/BW Reporting	PC Reporting
S-F120HA	[47]	≤0.001°/h; 1σ, 100 s	≤0.0002°/√h; method NR	±400°/s; BW ≥ 200 Hz, method NR	≤4 W, steady-state
S-F120HB	[47]	≤0.002°/h; 1σ, 100 s	≤0.0003°/√h; method NR	±400°/s; BW ≥ 200 Hz, method NR	≤4 W, steady-state
S-F120HC	[47]	≤0.003°/h; 1σ, 100 s	≤0.0004°/√h; method NR	±400°/s; BW ≥ 200 Hz, method NR	≤4 W, steady-state
GF120H-A	[38]	≤0.010°/h (1σ, 10 s); ≤0.004°/h (1σ, 100 s); two reported zero-bias stability limits	≤0.002°/√h; method NR	±500°/s; BW 100 Hz, model-specific documentation unavailable	6 W; model-specific documentation unavailable
EG-1300	[39]	≤0.010°/h; in-run, 25 °C, 1σ maximum	≤0.002°/√h; 25 °C	±300°/s; BW 500 Hz, −3 dB	5 W, maximum
DSP-1760	[41]	≤0.050°/h; 1σ typical, 25 °C	≤0.012°/√h; 25 °C	±490°/s; BW ≥ 440 Hz, −3 dB, housed	3 W typical; 6 W maximum, single-axis
EG-200	[42]	≤1.000°/h; in-run, 25 °C	≤0.040°/√h; 25 °C	±300°/s; BW 300 Hz, −3 dB/−90°	<1.3 W, room temperature
DSP-3000	[43]	≤1.000°/h; 25 °C, 1σ, digital	≤0.067°/√h; 25 °C, digital	±375°/s; BW ≥ 440 Hz, −3 dB	1.25 W typical; 3 W maximum
VG103SD	[45]	1.000°/h; RMS; averaging conditions NR	0.015°/√h; method NR	400°/s; BW 600 Hz, method NR	0.5 W; type NR
VG1703	[44]	1.000°/h; RMS; Allan variance minimum	0.008°/√h; white-noise PSD	130°/s; BW 1000 Hz, second-order LPF	0.5 W, +5 V
VG910F1	[46]	1.000°/h; RMS; Allan variance minimum, day-to-day	0.020°/√h; method NR	400°/s; BW 1000 Hz, third-order LPF	0.5 W typical, 20 °C

Note: BS—Bias Stability; ARW—Angle Random Walk; MIR—Maximum Input Rate; BW—Bandwidth; PC—Power Consumption; NR—Not Reported. The table summarizes the specification definitions and operating conditions available in the examined technical documentation. Values preceded by ≤ or ≥ represent manufacturer-reported specification limits rather than independently measured values. Values reported without inequality symbols may also represent manufacturer-defined nominal, typical, or other specification values rather than independently measured observations. The characteristics were not obtained under a common experimental protocol, and compliance with IEEE Std 952-2020 was not independently established.

**Table 6:** Normalized evaluation criteria of the investigated FOGs.

Gyroscope	BS_n_	ARW_n_	MIR_n_	BW_n_	TRW_n_	PC_n_
S-F120HA	1.000	1.000	0.730	0.111	1.000	0.364
S-F120HB	0.999	0.999	0.730	0.111	1.000	0.364
S-F120HC	0.998	0.997	0.730	0.111	1.000	0.364
GF120H-A	0.997	0.973	1.000	0.000	0.000	0.000
EG-1300	0.991	0.973	0.459	0.444	0.500	0.182
DSP-1760	0.951	0.823	0.973	0.378	1.000	0.545
EG-200	0.000	0.404	0.459	0.222	1.000	0.855
DSP-3000	0.000	0.000	0.662	0.378	1.000	0.864
VG103SD	0.000	0.778	0.730	0.556	0.500	1.000
VG1703	0.000	0.883	0.000	1.000	0.500	1.000
VG910F1	0.000	0.704	0.730	1.000	0.500	1.000

**Table 7:** Group scores, FOGPI values, and ranking of the investigated FOGs under the baseline weighting configuration.

Gyroscope	Accuracy	Dynamic Performance	Operating Temperature Capability	Power Requirement	FOGPI	Rank
DSP-1760	0.887	0.675	1.000	0.545	0.8170	1
S-F120HA	1.000	0.420	1.000	0.364	0.7915	2
S-F120HB	0.999	0.420	1.000	0.364	0.7908	3
S-F120HC	0.998	0.420	1.000	0.364	0.7902	4
EG-1300	0.982	0.452	0.500	0.182	0.6972	5
GF120H-A	0.985	0.500	0.000	0.000	0.6175	6
VG910F1	0.352	0.865	0.500	1.000	0.5671	7
VG103SD	0.389	0.643	0.500	1.000	0.5303	8
VG1703	0.442	0.500	0.500	1.000	0.5208	9
EG-200	0.202	0.341	1.000	0.855	0.4217	10
DSP-3000	0.000	0.520	1.000	0.864	0.3663	11

**Table 8:** Comparison of FOGPI scores and rankings under linear and logarithmic normalization.

Gyroscope	Linear FOGPI	Rank	Log FOGPI	Rank
S-F120HA	0.791469	2	0.791469	1
S-F120HB	0.790844	3	0.748948	2
S-F120HC	0.790220	4	0.721904	3
GF120H-A	0.617513	6	0.475820	6
EG-1300	0.697181	5	0.523828	5
DSP-1760	0.816965	1	0.555757	4
EG-200	0.421713	10	0.342844	11
DSP-3000	0.366356	11	0.366356	10
VG103SD	0.530271	8	0.400014	8
VG1703	0.520808	9	0.391383	9
VG910F1	0.567114	7	0.443200	7

**Table 9:** Comparison of baseline, fixed-linear, and fixed-logarithmic FOGPI scores.

Gyroscope	Original FOGPI	Fixed Linear	Fixed Logarithmic
S-F120HA	0.791469	0.749727	0.674914
S-F120HB	0.790844	0.749452	0.645188
S-F120HC	0.790220	0.749177	0.625972
GF120H-A	0.617513	0.715148	0.531478
EG-1300	0.697181	0.754998	0.551581
DSP-1760	0.816965	0.770223	0.493036
EG-200	0.421713	0.652152	0.360162
DSP-3000	0.366356	0.611960	0.368869
VG103SD	0.530271	0.767715	0.448659
VG1703	0.520808	0.801482	0.487659
VG910F1	0.567114	0.805202	0.488248

Note: All calculations use the baseline weighting coefficients and updated technical specifications. Fixed-reference scores are calculated using the reference bounds specified in Section 2.7.

**Table 10:** Sensitivity of the FOGPI ranking to ±20% variations in functional-group weights.

Varied Group	Variation	Accuracy	Dynamic Performance	Operating Temperature Capability	Power Requirement	Highest-Ranked FOG	Spearman *ρ*	Kendall τ
Baseline	-	0.500	0.250	0.150	0.100	DSP-1760	1.000	1.000
Accuracy	−20%	0.400	0.300	0.180	0.120	DSP-1760	0.973	0.927
Accuracy	+20%	0.600	0.200	0.120	0.080	S-F120HA	0.936	0.855
Dynamic Performance	−20%	0.533	0.200	0.160	0.107	DSP-1760	1.000	1.000
Dynamic Performance	+20%	0.467	0.300	0.140	0.093	DSP-1760	1.000	1.000
TRW	−20%	0.518	0.259	0.120	0.104	DSP-1760	1.000	1.000
TRW	+20%	0.482	0.241	0.180	0.096	DSP-1760	1.000	1.000
Power Consumption	−20%	0.511	0.256	0.153	0.080	DSP-1760	1.000	1.000
Power Consumption	+20%	0.489	0.244	0.147	0.120	DSP-1760	1.000	1.000

**Table 11:** Mission-oriented AHP weighting scenarios and resulting FOGPI rankings.

Scenario	A	D	T	P	CR	Top-Ranked FOGs (FOGPI)
Baseline	0.5000	0.2500	0.1500	0.1000	—	DSP-1760 (0.8170)
Long-duration INS	0.6143	0.1972	0.1186	0.0699	0.0168	S-F120HA (0.8413); S-F120HB (0.8405); S-F120HC (0.8397)
UAV/Tactical	0.3936	0.3936	0.1375	0.0753	0.0015	DSP-1760 (0.7936); S-F120HA (0.7240); S-F120HB (0.7235)
Compact Robotics	0.1699	0.2844	0.0729	0.4729	0.0189	VG910F1 (0.8150); VG103SD (0.7582); VG1703 (0.7265)

**Table 12:** Global weight-space rank acceptability analysis.

Gyroscope	Global P (1), %	Global P (Top 3), %	Baseline P (1), %	Baseline P (Top 3), %
S-F120HA	11.71	52.23	12.61	99.99
S-F120HB	0.00	44.49	0.00	99.78
S-F120HC	0.00	11.35	0.00	11.24
GF120H-A	0.01	0.40	0.00	0.00
EG-1300	0.00	0.03	0.00	0.00
DSP-1760	50.72	70.05	87.39	88.76
EG-200	2.09	11.01	0.00	0.00
DSP-3000	4.90	18.45	0.00	0.00
VG103SD	0.00	31.72	0.00	0.01
VG1703	2.28	15.68	0.00	0.00
VG910F1	28.30	44.58	0.00	0.22

**Table 13:** Spearman rank-correlation matrix for the FOG evaluation criteria.

Criterion	BS	ARW	MIR	BW	TRW	PC
BS	1.000	0.922	−0.441	0.759	−0.267	−0.806
ARW	0.922	1.000	−0.280	0.545	−0.064	−0.664
MIR	−0.441	−0.280	1.000	−0.499	−0.054	0.355
BW	0.759	0.545	−0.499	1.000	−0.336	−0.779
TRW	−0.267	−0.064	−0.054	−0.336	1.000	0.026
PC	−0.806	−0.664	0.355	−0.779	0.026	1.000

**Table 14:** Comparison of baseline and CRITIC-derived criterion weights.

Criterion	Baseline Weight	CRITIC Weight
BS	0.2500	0.2317
ARW	0.2500	0.1370
MIR	0.1250	0.1242
BW	0.1250	0.1702
TRW	0.1500	0.1532
PC	0.1000	0.1837
**Total**	1.0000	1.0000

**Table 15:** Comparison of baseline and CRITIC-weighted FOGPI scores and rankings.

Gyroscope	Baseline FOGPI	Rank	CRITIC-FOGPI	Rank
DSP-1760	0.816965	1	0.771712	1
S-F120HA	0.791469	2	0.698309	2
S-F120HB	0.790844	3	0.697872	3
S-F120HC	0.790220	4	0.697435	4
EG-1300	0.697181	5	0.605671	6
GF120H-A	0.617513	6	0.488547	9
VG910F1	0.567114	7	0.617471	5
VG103SD	0.530271	8	0.552104	7
VG1703	0.520808	9	0.551471	8
EG-200	0.421713	10	0.460447	10
DSP-3000	0.366356	11	0.458371	11

Note: CRITIC weights were calculated using Pearson correlations and standard deviations of the unrounded, linearly normalized criteria. Both weighting configurations were applied to the same decision matrix.

**Table 16:** Leave-one-out sensitivity of the FOGPI ranking to changes in the comparison dataset.

Removed FOG	Highest-Ranked Remaining FOG	Spearman *ρ*	Kendall τ
S-F120HA	DSP-1760	1.000	1.000
S-F120HB	DSP-1760	1.000	1.000
S-F120HC	DSP-1760	1.000	1.000
GF120H-A	DSP-1760	1.000	1.000
EG-1300	DSP-1760	1.000	1.000
DSP-1760	S-F120HA	1.000	1.000
EG-200	DSP-1760	1.000	1.000
DSP-3000	S-F120HA	0.915	0.822
VG103SD	DSP-1760	1.000	1.000
VG1703	DSP-1760	1.000	1.000
VG910F1	DSP-1760	1.000	1.000

**Table 17:** Monte Carlo sensitivity of FOGPI scores and rankings under independent uniform ±10% perturbations of the input specifications.

Gyroscope	Mean FOGPI	95% Scenario Interval	P (First), %	P (Top 3), %
S-F120HA	0.7311	0.6432–0.8013	19.85	64.25
S-F120HB	0.7306	0.6426–0.8006	19.59	63.76
S-F120HC	0.7300	0.6423–0.7998	19.24	63.46
GF120H-A	0.6423	0.5999–0.7112	0.11	4.61
EG-1300	0.6793	0.6072–0.7628	1.93	22.99
DSP-1760	0.7548	0.6672–0.8235	39.28	80.91
EG-200	0.3769	0.2832–0.4580	0.00	0.00
DSP-3000	0.3208	0.2294–0.3978	0.00	0.00
VG103SD	0.5261	0.4464–0.6151	0.00	0.00
VG1703	0.5173	0.4420–0.6032	0.00	0.00
VG910F1	0.5605	0.4804–0.6499	0.00	0.00

**Table 18:** Comparative ranking of the investigated FOGs using FOGPI, TOPSIS, and VIKOR.

Gyroscope	FOGPI	Rank	TOPSIS *C_i_*	Rank	VIKOR *Q_i_*	Rank
DSP-1760	0.8170	1	0.7785	5	0.0000	1
S-F120HA	0.7915	2	0.7814	2	0.1251	2
S-F120HB	0.7908	3	0.7812	3	0.1258	3
S-F120HC	0.7902	4	0.7810	4	0.1265	4
EG-1300	0.6972	5	0.7982	1	0.1446	5
GF120H-A	0.6175	6	0.7391	6	0.4310	6
VG910F1	0.5671	7	0.5644	9	0.7772	7
VG103SD	0.5303	8	0.5782	8	0.8181	8
VG1703	0.5208	9	0.6189	7	0.8286	9
EG-200	0.4217	10	0.3539	10	0.9386	10
DSP-3000	0.3664	11	0.1902	11	1.0000	11

**Table 19:** Rank correlations among FOGPI, TOPSIS, and VIKOR.

Compared Methods	Spearman *ρ*	Kendall τ
FOGPI–TOPSIS	0.818	0.636
FOGPI–VIKOR	1.000	1.000
TOPSIS–VIKOR	0.818	0.636

**Table 20:** Operating-temperature specifications and admissibility under two illustrative thermal scenarios.

Gyroscope	Tmin (°C)	Tmax (°C)	TRW (°C)	Cold	Upper
S-F120HA	−45	+70	115	Yes	No
S-F120HB	−45	+70	115	Yes	No
S-F120HC	−45	+70	115	Yes	No
GF120H-A	−40	+65	105	No	No
EG-1300	−40	+70	110	No	No
DSP-1760	−40	+75	115	No	Yes
EG-200	−40	+75	115	No	Yes
DSP-3000	−40	+75	115	No	Yes
VG103SD	−40	+70	110	No	No
VG1703	−40	+70	110	No	No
VG910F1	−40	+70	110	No	No

Note: Cold—extended cold operation (−45 to +65 °C); Upper—extended upper-temperature operation (−40 to +75 °C). Yes indicates that the manufacturer-specified operating-temperature interval fully covers the required interval. The scenarios are illustrative and do not represent standardized environmental qualification requirements.

**Table 21:** Application-specific admissibility screening and FOGPI ranking results.

Scenario	Admissible FOGs	Number	Highest-Ranked FOG	FOGPI
High-accuracy INS	S-F120HA, S-F120HB, S-F120HC, GF120H-A, EG-1300	5	S-F120HA	0.791469
Compact UAV	EG-200, VG103SD, VG910F1	3	VG910F1	0.567114

Note: Admissibility was determined using the mandatory requirements defined in Section 2.13. FOGPI scores were calculated using the original baseline normalization and weighting configuration without recalculating normalization bounds after screening. The scenarios represent illustrative engineering requirements.

## Data Availability

The original contributions presented in this study are included in the article. Further inquiries can be directed to the corresponding authors.

## Abbreviations

AHP	Analytic Hierarchy Process
ARW	Angle Random Walk
BS	Bias Stability
BW	Bandwidth
MIR	Maximum Input Rate
FOG	Fiber-Optic Gyroscope
FOGPI	Fiber Optic Gyroscope Performance Index
INS	Inertial Navigation System
MCDM	Multi-Criteria Decision-Making
PC	Power Consumption
TRW	Operating Temperature Range Width
TOPSIS	Technique for Order Preference by Similarity to Ideal Solution
VIKOR	VIseKriterijumska Optimizacija I Kompromisno Resenje

## Appendix A. AHP Pairwise Comparison Matrices and Consistency Assessment

### Appendix A.1. Long-Duration Inertial Navigation Scenario

The long-duration INS scenario assigns the greatest importance to Accuracy, reflecting the assumed priority of limiting bias-related and stochastic-noise contributions to accumulated navigation errors during autonomous operation.

**Table A1:** AHP pairwise comparison matrix for the long-duration INS scenario.

Criterion	A	D	T	P
A	1	4	5	7
D	1/4	1	2	3
T	1/5	1/2	1	2
P	1/7	1/3	1/2	1

The normalized principal right eigenvector yields the following group weights: Accuracy = 0.614345, Dynamic Performance = 0.197168, Operating Temperature Capability = 0.118611, and Power Requirement = 0.069876. The principal eigenvalue is 4.045290, corresponding to a consistency ratio of CR = 0.016774.

### Appendix A.2. UAV/Tactical Navigation Scenario

The UAV/tactical navigation scenario assigns equal importance to Accuracy and Dynamic Performance to reflect the combined requirements of maintaining navigation accuracy and measuring rapid angular motion.

**Table A2:** AHP pairwise comparison matrix for the UAV/tactical navigation scenario.

Criterion	A	D	T	P
A	1	1	3	5
D	1	1	3	5
T	1/3	1/3	1	2
P	1/5	1/5	1/2	1

The normalized principal right eigenvector yields the following group weights: Accuracy = 0.393618, Dynamic Performance = 0.393618, Operating Temperature Capability = 0.137474, and Power Requirement = 0.075290. The principal eigenvalue is 4.004158, corresponding to a consistency ratio of CR = 0.001540.

### Appendix A.3. Compact Robotic Systems Scenario

The compact robotic systems scenario assigns the greatest importance to Power Requirement, followed by Dynamic Performance, representing the assumed priorities of energy-constrained mobile robotic platforms.

**Table A3:** AHP pairwise comparison matrix for the compact robotic systems scenario.

Criterion	A	D	T	P
A	1	1/2	3	1/3
D	2	1	4	1/2
T	1/3	1/4	1	1/5
P	3	2	5	1

The normalized principal right eigenvector yields the following group weights: Accuracy = 0.169901, Dynamic Performance = 0.284378, Operating Temperature Capability = 0.072859, and Power Requirement = 0.472862. The principal eigenvalue is 4.051110, corresponding to a consistency ratio of CR = 0.018929.

### Appendix A.4. Summary of AHP Weights and Consistency Assessment

The resulting weighting vectors, principal eigenvalues, consistency indices, and consistency ratios for the three mission-oriented scenarios are summarized in Table A4.

**Table A4:** AHP-derived weighting coefficients and consistency assessment for the three mission-oriented scenarios.

Scenario	*A*	*D*	*T*	*P*	*λ*_max_	CI	CR
Long-duration INS	0.614345	0.197168	0.118611	0.069876	4.045290	0.015097	0.016774
UAV/Tactical	0.393618	0.393618	0.137474	0.075290	4.004158	0.001386	0.001540
Compact Robotics	0.169901	0.284378	0.072859	0.472862	4.051110	0.017037	0.018929

Note: *A*—Accuracy; *D*—Dynamic Performance; *T*—Operating Temperature Capability; *P*—Power Requirement; *λ*_max_—principal eigenvalue; CI—Consistency Index; CR—Consistency Ratio. All values are rounded for presentation.

All three scenarios satisfy the adopted consistency threshold of *CR* < 0.10, indicating acceptable internal consistency of the assumed pairwise judgments. However, consistency does not establish the objective validity of the engineering preferences or the suitability of the resulting weights for specific operational missions.

The weighting coefficients presented in this appendix correspond to the mission-oriented FOGPI evaluation reported in Section 3.4.2. The calculations were performed using unrounded eigenvector weights, while the values reported in the main text were rounded for presentation.
